# Interactive effects of salinity, redox, and colloids on greenhouse gas production and carbon mobility in coastal wetland soils

**DOI:** 10.1371/journal.pone.0316341

**Published:** 2024-12-30

**Authors:** Nicholas D. Ward, Madison Bowe, Katherine A. Muller, Xingyuan Chen, Qian Zhao, Rosalie Chu, Zezhen Cheng, Thomas W. Wietsma, Ravi K. Kukkadapu

**Affiliations:** 1 Coastal Sciences Division, Pacific Northwest National Laboratory, Sequim, Washington, United States of America; 2 School of Oceanography, University of Washington, Seattle, Washington, United States of America; 3 Earth Systems Science Division, Pacific Northwest National Laboratory, Richland, Washington, United States of America; 4 Atmospheric Science & Global Change Division, Pacific Northwest National Laboratory, Richland, Washington, United States of America; 5 Environmental Molecular Sciences Division, Pacific Northwest National Laboratory, Richland, Washington, United States of America; ICAR National Rice Research Institute, INDIA

## Abstract

Coastal wetlands, including freshwater systems near large lakes, rapidly bury carbon, but less is known about how they transport carbon either to marine and lake environments or to the atmosphere as greenhouse gases (GHGs) such as carbon dioxide and methane. This study examines how GHG production and organic matter (OM) mobility in coastal wetland soils vary with the availability of oxygen and other terminal electron acceptors. We also evaluated how OM and redox-sensitive species varied across different size fractions: particulates (0.45–1μm), fine colloids (0.1–0.45μm), and nano particulates plus truly soluble (<0.1μm; NP+S) during 21-day aerobic and anaerobic slurry incubations. Soils were collected from the center of a freshwater coastal wetland (FW-C) in Lake Erie, the upland-wetland edge of the same wetland (FW-E), and the center of a saline coastal wetland (SW-C) in the Pacific Northwest, USA. Anaerobic methane production for FW-E soils were 47 and 27,537 times greater than FW-C and SW-C soils, respectively. High Fe^2+^ and dissolved sulfate concentrations in FW-C and SW-C soils suggest that iron and/or sulfate reduction inhibited methanogenesis. Aerobic CO_2_ production was highest for both freshwater soils, which had a higher proportion of OM in the NP+S fraction (64±28% and 70±10% for FW-C and FW-E, respectively) and organic C:N ratios reflective of microbial detritus (5.3±5.3 and 5.3±7.0 for FW-E and FW-C, respectively) compared to SW-C, which had a higher fraction of particulate (58±9%) and fine colloidal (19±7%) OM and organic C:N ratios reflective of vegetation detritus (11.4 ± 1.7). The variability in GHG production and shifts in OM size fractionation and composition observed across freshwater and saline soils collected within individual and across different sites reinforce the high spatial variability in the processes controlling OM stability, mobility, and bioavailability in coastal wetland soils.

## Introduction

Vegetated coastal systems play a disproportionate role in carbon cycling compared to their land cover area, as they occupy only 0.07–0.22^%^ of Earth’s surface, yet account for approximately 10^%^ of the net residual land carbon sink [1]. The magnitude of this important coastal carbon sink is driven primarily by high soil carbon burial rates relative to terrestrial and marine ecosystems [2] and is modified by lateral and vertical export from the system. Lateral export of organic and inorganic carbon from coastal ecosystems to open water environments may add to the total amount of carbon sequestered [3] if the exported carbon persists in the ocean without being returned to the atmosphere [4]. In contrast, a fraction of a coastal ecosystem’s carbon sink is offset by ecosystem emissions of greenhouse gases (GHGs) such as carbon dioxide (CO_2_), methane (CH_4_) and nitrous oxide to the atmosphere [5, 6]. The extent to which carbon is either stored in coastal soils, transported laterally to the ocean, or emitted the atmosphere as GHGs is likely controlled by a wide variety of hydrological, ecological, microbial, and geophysical factors; we currently lack a predictive understanding of these complex and potentially changing controls [7, 8].

It is well known that soil is a dynamic organic matter (OM) reservoir that is a major component of the global carbon cycle [9, 10], but less is known about the factors that mediate lateral export out of the soil system [11, 12], particularly along the coast [13, 14]. Substantial progress has been made over the last several decades to quantify the amount and composition of carbon exported laterally from coastal ecosystems [15–17], but the physiochemical controls on carbon export are only beginning to be elucidated [18]. The mobile (i.e., aqueous) phase of soil OM can transition between particulate, colloidal, and dissolved forms and the interaction of these forms with mineral surfaces under specific environmental conditions plays an important role in either mobilizing or stabilizing soil OM [19]. The complexities of how soil OM fractionates across different size fractions, and the relative reactivity of these fractions, is not currently considered in our understanding of lateral carbon export from coastal wetlands [20–22].

In addition to export out of wetland soils, OM is decomposed in soils yielding GHG release, the rate of which is also influenced by a combination of factors including microbial community composition and enzymatic activity, soil aggregation, soil mineralogy, and environmental variables [23, 24]. Reducing conditions generally lower CO_2_ production rates via a hypothesized mechanism referred to as the “enzymic latch,” in which anoxia inhibits the activity of phenol oxidase enzymes [25]. CH_4_ production, with some exceptions [26], generally relies on anoxic conditions, and the magnitude of CH_4_ production depends on competition with other terminal electron acceptors such as nitrate, sulfate, iron, and manganese [27]. Coastal ecosystems both contain and are exposed to all of these competing electron acceptors [5], and, likewise, experience fluctuating redox states [28], presenting a large challenge for constraining current and future coastal ecosystem GHG cycling dynamics. It has been proposed that salinity exposure is a dominant control on coastal GHG emissions due. Sulfate found in seawater has been associated with increased sulfate reduction rates [29] and thus increased CO_2_ emissions from carbon mineralization [30] and decreased methanogenesis [31], but this has not been consistently demonstrated across studies [32].

The heterogeneous nature of soil environments further complicates the interpretation of the biogeochemical behaviors described above. Biogeochemical processes can be highly influenced by heterogenous characteristics such as soil microsites [33], differing soil pore sizes [34], microbial community diversity [35], and soil aggregation dynamics [36] among other factors. The size distribution of water-soluble OM and redox-sensitive elements in soil porewaters is another understudied factor that might play a key role in determining the mobility and/or reactivity of OM in soils. Laboratory characterizations of OM often focus on dissolved organic carbon (DOC), operationally defined as the carbon that passes through filters ranging in pore size from 0.2 to 0.7 μm; this practice has been recently questioned due to the resulting overestimation of truly soluble DOC via the inclusion of colloids [19, 37, 38]. The importance of understanding the role of colloids in soil carbon cycling stems in part from their highly reactive surfaces that permit binding to OM [39]. Thus, understanding how the biogeochemical behavior of colloidal size fractions varies under different environmental and mineralogical conditions is central to constraining the mechanisms underlying soil OM transport and transformation.

Given the array of factors influencing carbon cycling in coastal wetlands, this study evaluates a subset of potential drivers of either mobilization or remineralization of coastal wetland soil carbon to CO_2_ or CH_4_—exposure to salinity, redox state, terminal electron acceptor abundance, OM size fractionation, and flooding history. Using soils collected from three different freshwater and saline wetland settings, we conducted 21-day incubations in aerobic and anaerobic conditions to tested how oxygen availability, soil origin, and inundation history influence the evolution of GHG production, bulk chemical properties, redox sensitive species, and biodegradation of OM across three size fractions: nano particulates plus truly soluble OM (NP+S; < 0.1 μm), fine colloids (FC; 0.1–0.45 μm), and particulates (P; 0.45–1 μm). We hypothesized that anaerobic methane production would be outcompeted by iron or sulfate reduction for soils with exposure to high levels of either aqueous or mineral-derived sulfate and iron, and that the relative proportion of soluble versus mineral-associated colloidal and particulate OM would be an important factor mediating aerobic respiration.

## Materials and methods

### Soil collections

Three distinct sets of soil samples were collected from freshwater and saline coastal wetlands to test the above hypothesis. High levels of seawater exposure throughout the year at the saline wetland allowed us to compare the impacts of sulfate and other ions associated with salinity on both GHG production and OM solubility and size fractionation compared to the freshwater sites with no seawater exposure. Two samples collected at frequently and infrequently flooded locations along the same freshwater wetland allowed us to further examine the impact of flood history on terminal electron acceptor abundance and associated changes in GHG production and OM size fractionation. The diverse geochemistry of the studied soils also provided an opportunity to use results from the experiments described in this study to test the skill of new reactive transport model developments across a wide range of OM abundance/compositions and terminal electron acceptor availability [40].

The two freshwater (FW) surface soil samples were collected from a wetland located near the outlet of Old Woman Creek into Lake Erie (Huron, Ohio, United States). These samples were collected at the wetland center (i.e., in the center of the wetland, FW-C; 41.37613787°, -82.50754702°) and at the upland-wetland edge (i.e., near the border between where wetland vegetation starts transitioning to upland vegetation, FW-E; 41.37590722, -82.5071329°). FW-C soils are characterized primarily as frequently flooded silty fluvaquents; FW-E soils are occasionally flooded and characterized primarily as Holly silt loam [41]. In general, the freshwater wetland site is characterized by surface water with salinity generally between 0.1 and 0.3 PSU, and dissolved oxygen (DO) ranging from 5–15 mg/L [42]. At the time of soil collection on 12/9/2021, porewater DO was 10.5 mg/L at the less frequently flooded FW-E site and 1.5 mg/L at the more frequently flooded FW-C site, measured using a YSI Pro Plus multiparameter sonde connected to a porewater sipper rod (M.H.E. products).

A third sample was collected at the center of a saline wetland (SW-C) from the floodplain of Beaver Creek (46.905938°, -123.978047°), a tidally influenced first-order tributary draining into Johns River, which flows into the Grays Harbor estuary in Washington state. Beaver Creek floodplain soils are characterized as hydric Ocosta silty clay loam [41] and soil texture is primarily silty clay, but ranges from sandy clay loam to clay [35]. Groundwater in this floodplain is generally anaerobic and has salinities between 15–30 PSU during dry periods and 5–20 PSU during wet periods [43]. Detailed site information is described by Yabusaki et al. [44]. When the soil was collected on 1/25/2022, porewater DO was 2.2 mg/L and salinity was 3.6 PSU. All soil samples were collected at approximately 30 cm depth and were stored in sealed bags at 4°C prior to incubation.

### Incubations, soil analyses, and greenhouse gas analyses

Prior to soil incubations, the soils were conditioned at room temperature in a Coy anaerobic chamber at O_2_ levels below 20 ppm for four days. Vegetation and rocks were removed by hand. A subsample of each soil was dried in aerobic conditions for bulk characterization of total C (TC), total nitrogen (TN), and total sulfur (TS) measured on an Elementar vario EL Elemental Analyzer (Figs 3–5 in S1 Text); percent moisture was also measured. TC, TN, and TS were also measured at the end of the incubations on solid soils remaining in the bottom of reactors. Incubations were conducted in triplicate combusted 1 L glass bottles with air-tight caps equipped with two luer valves that allowed for sampling and flushing of the bottle’s headspace.

To initiate the anerobic incubations, field moist soil was suspended in deoxygenated de-ionized water in the 1 L microcosms leaving a headspace of ~250 mL that was purged with N_2_. After shaking the bottles for 5 min, triplicate bottles were destructively sampled for size-fractionated chemical analyses (see section 2.3) for the initial timepoint (referred to as “pre-incubation”). Aerobic incubations were initiated by adding water with ambient O_2_ levels, purging the headspace with room air, shaking, then destructively sampling a subset of the bottles for chemical analyses. The soil:water ratio of the incubations (by mass) was 1:12.7 for FW-C soil, 1:16.5 for FW-E soil, and 1:11.0 for SW-C soil.

Incubations were carried out in the dark for 21 days at temperatures between 19–21°C at which point the incubated soil and the supernatant was destructively sampled for a variety of size-fractionated chemical analyses. The length of the incubation was chosen to ideally capture methanogenesis in at least one soil type following depletion of other competing terminal electron acceptors under anaerobic conditions and capture a plateau in aerobic CO_2_ production in at least one soil type following depletion of labile substrates so that these processes could be tested in models [40].

To both monitor GHG production in the bottles and maintain aerobic or anaerobic conditions during the incubations, headspace gas was sampled five days per week (i.e., Monday through Friday). Prior to gas sampling, incubations were shaken vigorously for 1–2 min to ensure that dissolved gasses in the headspace was equilibrated with dissolved gasses in the aqueous incubate with the headspace [45]. Headspace gas samples were collected using 60 mL syringes connected to one open luer valve while the other luer valve remained closed. After each gas sampling time point the bottles were purged with either N_2_ (anaerobic) or room air (aerobic) for several minutes with both luer valves open, then the bottles were sealed at ambient pressure. When purging the aerobic incubations with room air, one luer valve was left open to the lab atmosphere and the other valve was connected to a cavity ring-down spectrometer (CRDS; Picarro, G2508 Gas Concentration Analyzer) to determine when the headspace was fully equilibrated with room air. Likewise, this allowed us to quantify the amount of CO_2_ and CH_4_ added to the headspace between samplings when purging with room air.

With the collected 60 mL headspace samples, we first measured the partial pressures of headspace CO_2_ and CH_4_ on the CRDS with a flow limiter as described by Ward et al. [46]. Oxygen content was also measured with an optical oxygen meter (Pyroscience, FireSting GO_2_) to confirm that conditions remained either oxic or anoxic for our two treatments. The concentration of CO_2_ and CH_4_ in the headspace and dissolved in incubates were calculated using Henry’s law, the ideal gas law, and temperature-dependent coefficients [47]. See Magen et al. [48] for relevant equations. Cumulative moles of each gas produced during incubations were determined by correcting for gas removed during sampling and added during headspace purging.

### Size-fractionated chemical analyses

At the start and end of incubations, liquid suspensions were divided into 50-mL centrifuge tubes and centrifuged to generate supernatants of three size fractions following Afsar et al. [19]:

1. A <1 μm supernatant, which contains a mixture of soluble OM (S; <10 kDa– 2.5 nm), mineral-associated nanoparticulate colloids (NP; 2.5 nm– 0.1 μm), mineral-associated fine colloids (FC; 0.1 μm-0.45 μm), and mineral-associated particulate organic matter (P; 0.45 μm– 1.0 μm), was collected by centrifuging for 6 min at 220 rcf.
2. A <0.45 μm supernatant, which contains a composite of soluble OM, nanoparticulate, and fine colloidal fractions, was collected by centrifuging for 8 min at 810 rcf. Note that although each of these fractions are present in < 0.45 μm filtered samples, this fraction is often erroneously referred to as soluble dissolved organic carbon).
3. A <0.1 μm supernatant, which contains nanoparticulate and soluble OM, was collected by centrifuging for 8 min at 22,040 rcf. Pre-incubation samples and anaerobic incubations were handled in an anaerobic chamber to maintain anoxia prior to analyses.

These three sets of size-fractionated samples were independent sub-samples collected from the triplicate incubation bottles as opposed to residues from subsequent centrifugation steps. Section 2.4 describes chemical concentrations for discrete size fractions (e.g., 0.45–1 μm) were calculated.

**Fig 1:**
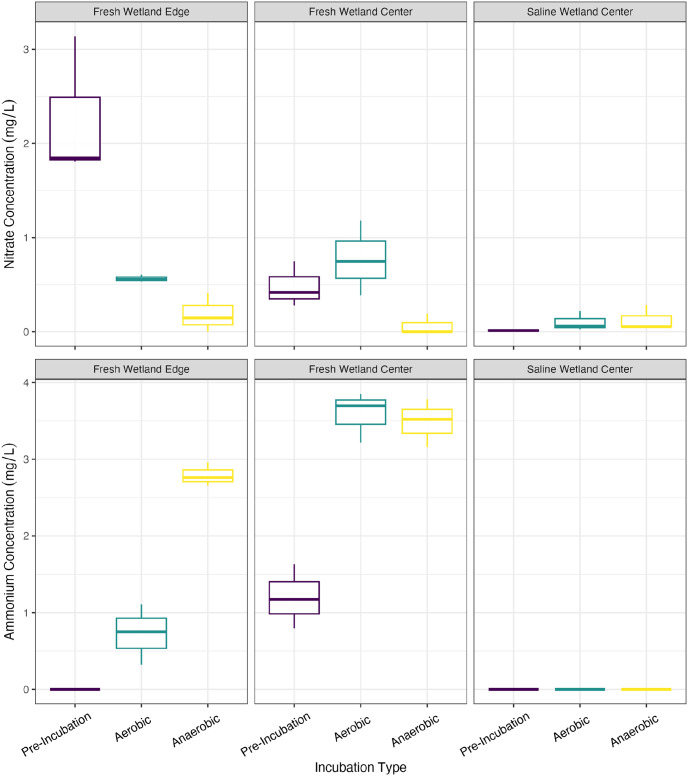
Nitrogen species. (Top) Nitrate and (bottom) ammonium concentrations in samples filtered to 0.2 μm pre- and post-incubation. The instrumentation used to measure nitrate did not allow us to analyze by colloidal size fraction without compromising the instrument.

**Fig 2:**
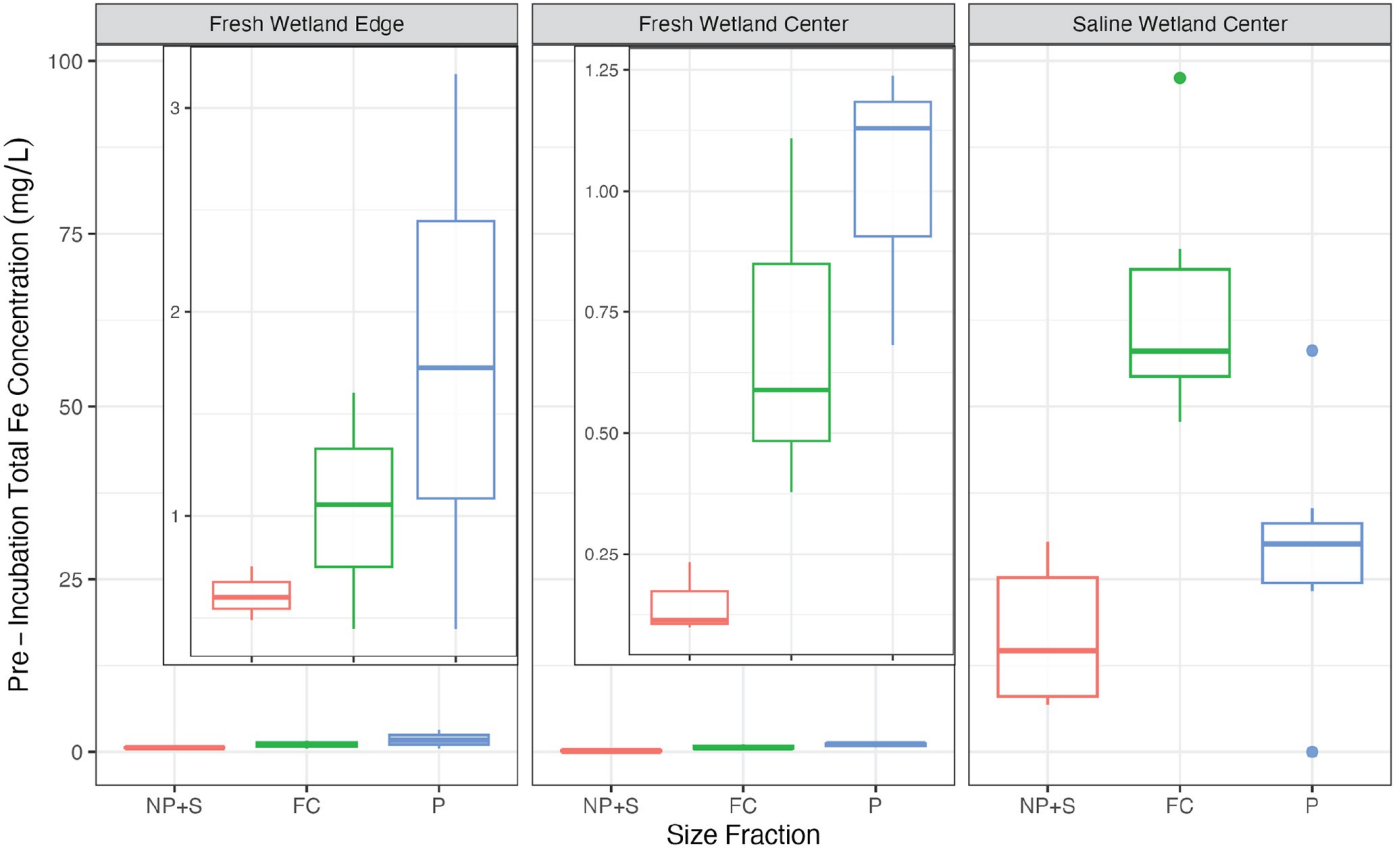
Total iron. Concentrations of total iron across size fractions prior to incubation. It was not possible to analyze post-incubation samples due to logistical constraints. Insets in the left and middle plots show a zoomed in y-axis.

To provide context of the physiochemical conditions each soil experienced during the incubations, we measured pH (NBS scale, DGi117 sensor, Mettler Toledo), oxidation-reduction potential (ORP; InLab Redox Flow sensor, Mettler Toledo), and alkalinity (Figs 1, 2 in S1 Text) on both size-fractionated (i.e., <1 and bulk (i.e., unfiltered) pre-incubation and incubated samples on an automatic titrator (Mettler-Toledo, T7 Excellence) following methods from Myers-Pigg et al. (2023). Alkalinity was measured by titrating samples with 0.02 N HCl to an endpoint of pH 4.00, following standard United States Geological Survey (USGS) procedures [49]. Measurements were made while stirring the samples with a magnetic stir bar to keep colloids in suspension and pH and ORP sensors were calibrated with three-point NBS buffers and ZoBell’s solution, respectively, before each analytical run and checked for drift every five samples.

The concentration of a variety of redox-sensitive elements were measured to understand how the abundance of different terminal electron acceptors may have competed with GHG production in the incubations. Sulfate, nitrate, ammonium, and nitrite concentrations were measured via ion chromatography (ThermoFisher, Dionex ICS-6000 HPIC). Due to instrument limitations, it was not possible to analyze by size fractions without damaging the instrument, so samples were filtered to 0.2 μm prior to analysis.

Size-fractionated aqueous samples were analyzed for ferrous iron (Fe^2+^) concentration by colorimetric assay (Thermo Scientific, FerroZine^™^ iron reagent) and total iron content and abundance via ICP-OES (Perkin Elmer, Optima 7300 DV). Ferrous iron concentration was analyzed using the ferrozine assay [50]. Briefly, 0.4 mL of well-mixed sample was added to 4 mL of 1 g/L ferrozine in 20 mM PIPES (piperazine-1,4-bis (2-ethanesulfonic acid)) buffer at pH 7 and shaken to homogenize. After five minutes, the solution was measured for the absorbance at 562 nm by an ultraviolet–visible spectrophotometer (UV–Vis) (Evolution 260 BIO, Thermo Scientific).

Finally, the concentration, size fractionation, and composition of aqueous phase organic matter was also measured to evaluate if carbon quality could potentially explain differences in GHG production. Aqueous TOC and TN concentrations were measured on size-fractionated and bulk samples on a Shimadzu TOC-L. Samples were stirred via a magnetic stir bar during analysis to ensure homogeneity. The concentration of total organic nitrogen (TON) was calculated by subtracting the concentration of nitrate, ammonium, and nitrite from TN. The ratio of TOC to TON was calculated to assess bulk geochemical differences in organic matter composition across size fractions. Samples were also characterized by 21T Fourier transform ion cyclotron resonance mass spectrometer (FTICR-MS) located at the Environmental Molecular Sciences Laboratory (Richland, WA) to molecularly characterize OM. Samples were randomized and directly infused into the FTICR-MS, after SPE clean-up [51], via an automated direct infusion cart [52]. Samples were measured in negative electrospray ionization (-ESI) polarity with technical replicates. All spectra were peak picked, internally calibrated and chemical formulae assigned using Formularity [53] considering only the presence of C, H, O, N, S and P.

### Data analyses

Chemical concentrations in each individual colloidal size fraction (e.g., <0.1 μm, 0.1–0.45, and 0.45–1 μm) were calculated by subtraction. TOC, TN, and Fe^2+^ concentrations in the largest size fraction (0.45–1 μm, or OM solely associated with particulates referred to as “P” throughout) were deduced by subtracting data from the <0.45 μm samples. Size-fractionated values for the medium-size fractions (0.1–0.45 μm, or solely fine colloids, referred to as “FC” throughout) were determined by subtracting values from the <0.1 μm (smallest size; composite of nanoparticulates and truly soluble OM, referred to as “NP+S” throughout) samples from those of the <0.45 μm samples. In the case of pH, ORP, and alkalinity it did not make conceptual sense to calculate the contribution of each size fraction via subtraction, therefore results are shown for the <1 μm, <0.45 μm, <0.1 μm, and bulk unfiltered samples without further calculations. Likewise, we did not attempt to calculate OM composition for each size fraction by subtraction for FT-ICR-MS data considering 1) solid phase extraction prior to analysis likely biases the results towards soluble OM and 2) this is not a quantitative measurement of concentrations.

All statistical analyses were performed in the statistical computing language R using R Studio version 2023.09.0+463 [54]. Incubation data was determined to not have a normal distribution based on visual inspection of density and QQ plots as well as the Shapiro Wilk’s test. Thus, we chose the non-parametric unpaired Wilcoxon rank sum test to evaluate the statistical significance of differences in measured parameters. Given the small number of replicates (in some cases n = 3 compared to n = 3), a one-sided Wilcoxon rank sum test was used to compare significant differences in chemical concentrations between pre- and post-incubation samples, incubation types, soil types, and size fractions. Reported p values represent comparisons of one group to another with the null hypothesis that the two sets of samples were not different from one another. Significant differences were considered to fall within a 95% confidence interval (i.e., p < 0.05). Pearson correlation was used to compare linearity in GHG production rates across the different experiments.

### Limitations

The comprehensive analytical measurements described above are very time consuming, which required us to prioritize what aspects of the experimental design to constrain. Wetland soil properties can vary substantially over relatively small spatial scales [55]. However, constraining such variability was not the goal of this study and, as such, replication was not performed at the field scale allowing us to instead replicate the incubation portion of our study. A single soil pit (roughly 0.5m in diameter) was dug at locations that visually exhibited the dominant aboveground features (e.g., vegetation type, ponding or lack thereof, etc.). Samples were collected from the face of each pit and homogenized prior to the incubations to ensure that there was limited variability across the experimental replicates. Our goal was to exclude field-scale spatial heterogeneity as a confounding factor for our aerobic and anaerobic lab incubation treatments. Previous studies have more specifically addressed spatial heterogeneity at both the Old Woman Creek [35] and Beaver Creek field sites [35, 56].

## Results

### Physiochemical conditions of the incubations

ORP of all size ranges of both freshwater wetland soils increased after being incubated under aerobic conditions for 21 days (Fig 1 in S1 Text). In contrast, ORP decreased, albeit to a smaller degree, in the saline samples under both aerobic and anaerobic conditions (Fig 1 in S1 Text). The observed increase in ORP for freshwater samples incubated under aerobic conditions was significant (p < 0.05) for all size fractions except for the bulk size fraction for FW-E, and the <0.1 μm size fraction for FW-C soils. The decrease in ORP under aerobic conditions observed for SW-C was significant (p > 0.05) for all size fractions except for the bulk unfiltered sample (p > 0.05). Under anaerobic conditions, ORP decreased for all the size ranges in FW-C and SW-C incubates but was slightly higher for the FW-E soils. These differences were statistically significant except for the <1 μm and <0.1 μm size fraction for FW-C soils and <0.45 μm size fraction for FW-E soils. ORP reached negative values in all but the smallest size fraction (<0.1 μm) for SW-C soils.

**Fig 3:**
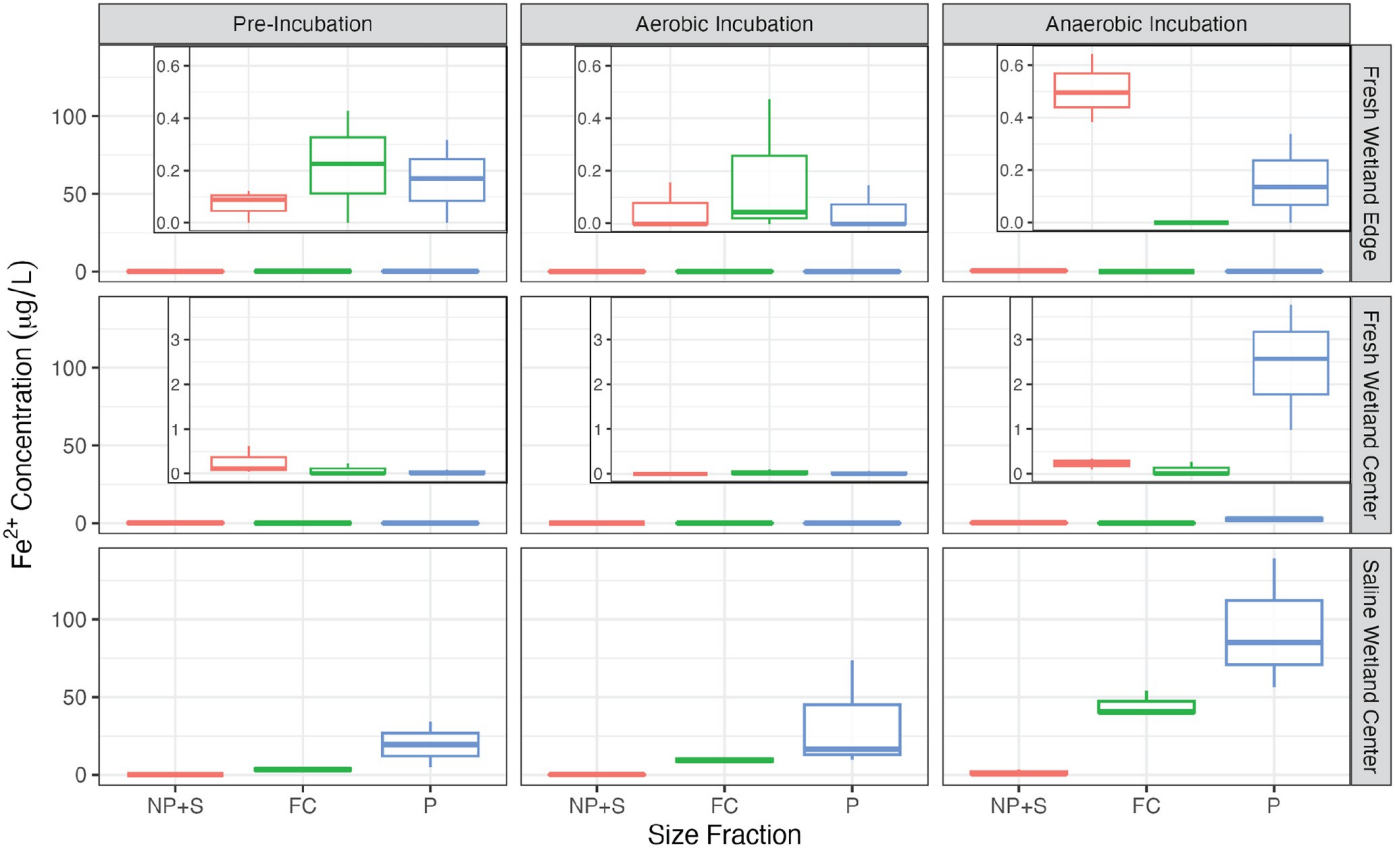
Reduced iron. Concentrations of Fe^2+^ across size fractions and incubation conditions. The smallest size fraction is nanoparticulates plus truly soluble material (NP+S; <0.1μm), followed by fine colloids (FC; 0.1–0.45μm), then particulates (P; 0.45–1μm). Insets in the left and middle plots show a zoomed in y-axis.

Alkalinity increased in all three soil types after being incubated under anaerobic conditions (p < 0.05) with the exception of the bulk and <0.1 μm size fraction for FW-C soils (p > 0.05). Alkalinity also increased in all soils and size fractions under aerobic conditions, but to a smaller extent (Fig 2 in S1 Text); these changes were statistically significant in all cases except for the bulk size fraction for FW-E soils and the bulk and <0.1 μm size fraction for FW-C soils (p > 0.05). pH was generally higher in the anaerobic incubations compared to aerobic and ranged from 6.0 to 7.7 across all size fractions, soil types, and incubation conditions (Fig 3 in S1 Text).

### Competing redox reactions

In this section we evaluate inorganic nitrogen species, sulfate, and iron as potential competing terminal electron acceptors for GHG production and redox cycles that may increase or decrease carbon mobility. The highest pre-incubation dissolved (<0.2 μm) nitrate concentrations were observed in the FW-E soils (2.26 ± 0.76 mg/L) compared to FW-C (0.48 ± 0.24 mg/L) and SW-C soils (0.01 ± 0.02 mg/L). Nitrate levels decreased significantly under both aerobic and anaerobic conditions for the FW-E soils as well as under anaerobic conditions for FW-C (p < 0.05; Fig 1). While there was slight variability in nitrate levels in the incubated FW-C aerobic soils and SW-C soils under both oxygen conditions, none of these changes were significant (p > 0.05). All three soils had barely detectable nitrate levels under anaerobic conditions (0.06–0.19 mg/L). Pre-incubation ammonium concentrations were 1.20 ± 0.42 mg/L in the FW-C soils and undetectable in the FW-E and SW-C soils (Fig 1). Ammonium levels increased significantly (p < 0.05) for both freshwater soils under both incubation conditions (Fig 1). Nitrite was undetectable in all samples.

Total iron at the beginning of the incubation was two orders of magnitude higher for SW-C soils compared to both freshwater soils (Fig 2; p < 0.05). For both freshwater soils, most of the iron was present as particulates and fine colloids with minimal nanoparticulate and soluble iron present (Fig 2). Fine colloids were the largest source of total iron for the SW-C soils and in contrast to the freshwater soils there was appreciable amounts of nanoparticulate and soluble iron prior to the incubations. Similar to total iron, there was minimal Fe^2+^ present in both freshwater samples prior to the incubation (Fig 3). Adding up all three size fractions (i.e., Fe^2+^ present in all forms < 1μm), the average initial Fe^2+^ concentration was 0.38 ± 0.25 μg/L for FW-E and 0.20 ± 0.17 μg/L for FW-C compared to an initial concentration of 18.4 ± 15.7 μg/L for SW-C. Particulates (i.e., 0.45–1.0 μm) were the dominant form of Fe^2+^ found in the pre-incubation samples for SW-C soils (73 ± 19% of the Fe^2+^ pool), whereas particulates and fine colloids contributed roughly equally for the FW-E soils, and Fe^2+^ was mostly present in the smallest NP+S fraction (67 ± 48%) for the pre-incubation FW-C soils (Fig 3).

For the FW-E soils, there was a slight increase in Fe^2+^ concentrations (all fractions, < 1μm) after incubating under anaerobic and a slight decrease under aerobic conditions (Fig 3), but these changes were not significant (p > 0.05). When considering each size fraction independently, there was a significant increase in the NP+S fraction under anaerobic conditions (p < 0.05), whereas there were no significant changes in the FC or P fractions or under aerobic conditions.

For the FW-C soils, Fe^2+^ concentrations (all fractions, < 1μm) similarly decreased under aerobic conditions (p < 0.05). In contrast to FW-E, Fe^2+^ concentrations increased to 2.70 ± 1.11 μg/L (all fractions, < 1μm) under anaerobic conditions for the FW-C soils, though this change was only significant when considering the particulate size fraction alone (p < 0.05). In the case of FW-C soils, there was a shift in the size distribution of Fe^2+^, with 84 ± 19% of the Fe^2+^ present under anaerobic conditions found in the particulate size fraction. SW-C soils had the highest Fe^2+^ levels initially and under both incubation conditions. In contrast to the other experiments, Fe^2+^ concentrations (all fractions, < 1μm) increased under both aerobic (42.6 ± 36.6 μg/L) and anaerobic conditions (140 ± 43 μg/L) but this change was only significant for anaerobic conditions (p < 0.05; Fig 3). The majority of Fe^2+^ was present as particulates under both aerobic (69 ± 16% of the Fe^2+^ pool) and anaerobic (65 ± 10%) conditions. The fine colloid fraction contained most of the remaining Fe^2+^ with less than 1% of the Fe^2+^ present in the smallest NP+S fraction.

**Fig 4:**
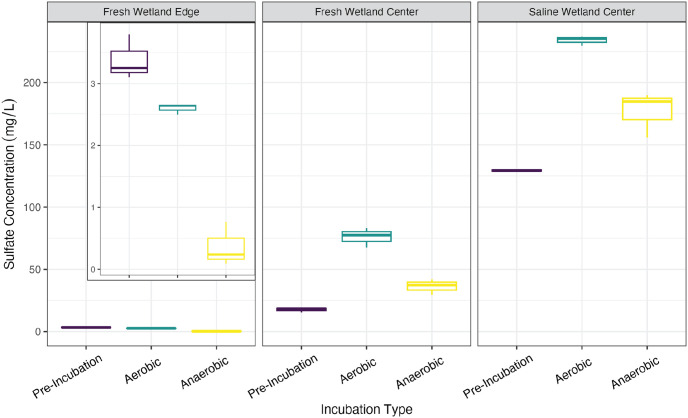
Sulfate levels. Sulfate concentrations in samples filtered to 0.2 μm pre- and post-incubation. The instrumentation used to measure sulfate did not allow us to analyze by colloidal size fraction without compromising the instrument. The insets in the left plot show a zoomed in y-axis.

Average initial aqueous sulfate concentrations from the SW-C soil (129 ± 1 mg/L) were substantially higher than for the FW-E and FW-C soils (3.38 ± 0.36 mg/L and 17.6 ± 1.9 mg/L, respectively), though this difference wasn’t statistically significant given the limited number of replicate samples for this analysis (p > 0.05; Fig 4). During the incubations, there was a strong scent of hydrogen sulfide detected while purging the headspace of the SW-C soils held under anaerobic conditions, suggesting active sulfate reduction was occurring. Although H_2_S was not directly measured, we did not smell H_2_S in the other soil incubations.

**Fig 5:**
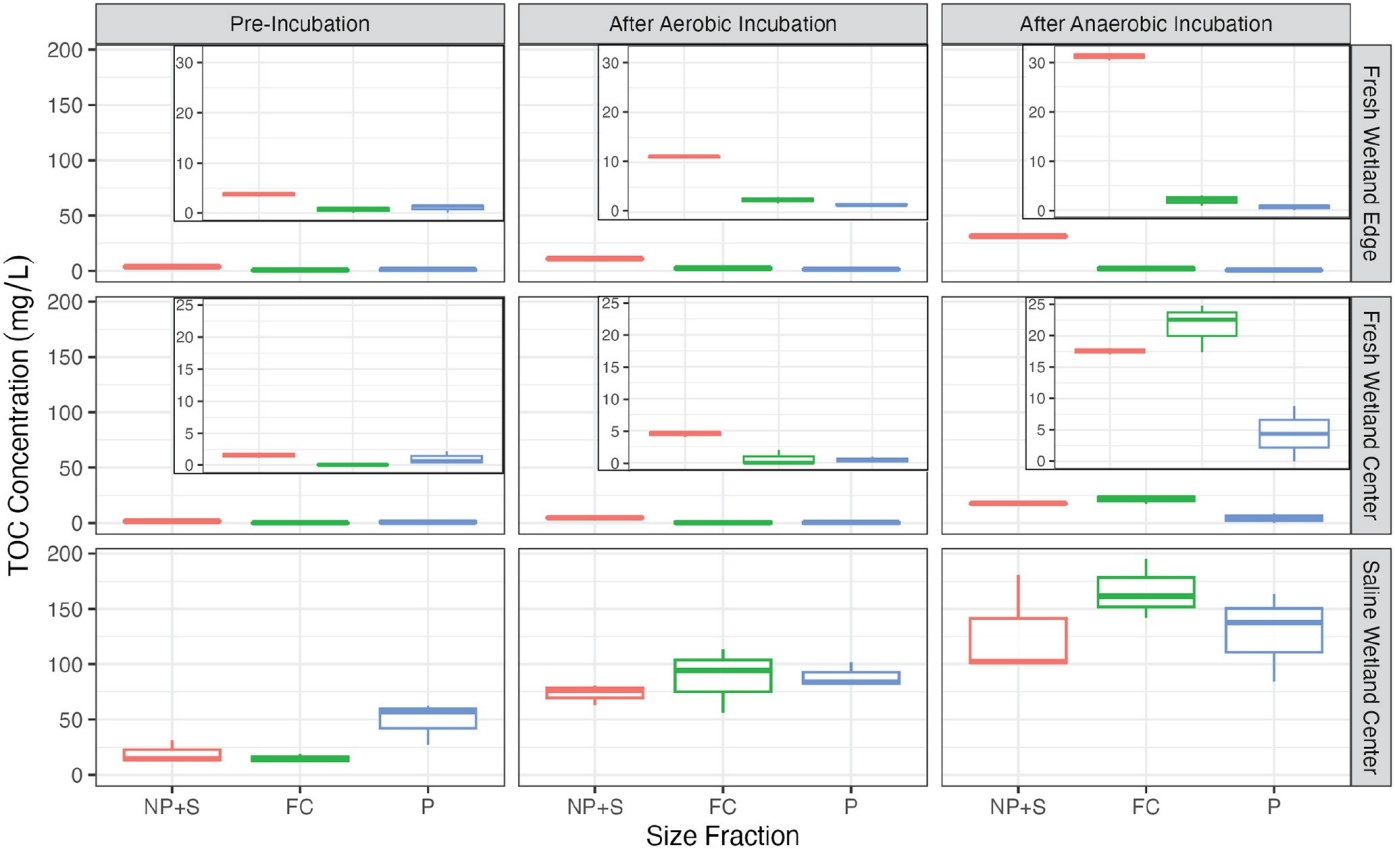
Organic carbon. Total organic carbon across size fractions and incubation conditions. The smallest size fraction is nanoparticulates plus truly soluble material (NP+S; <0.1μm), followed by fine colloids (FC; 0.1–0.45μm), then particulates (P; 0.45–1μm). Insets in the top and middle plots show a zoomed in y-axis.

For the SW-C soils, sulfate levels increased after the 21-day incubation under both aerobic (234 ± 4 mg/L) and anaerobic conditions (177 ± 18 mg/L), but once again this was not statistically significant due to limited replication (p > 0.05). Interestingly, sulfate levels also increased (p < 0.05) for the FW-C soils under both aerobic (76.0 ± 7.9 mg/L) and anaerobic conditions (36.2 ± 6.4 mg/L). Though in this case we did not detect an obvious presence of H_2_S. Both SW-C and FW-C anaerobic incubations ended with lower sulfate concentrations than their aerobic counterparts (57.1 mg/L and 39.7 mg/L lower for SW-C and FW-C soils, respectively), suggesting a solid phase source of sulfate. Corroborating this result, we also observed a decrease in solid phase sulfur content for the SW-C and FW-C soils under both incubation conditions, except for the FW-C anaerobic incubation (Fig 5 in S1 Text). FW-E on the other hand had lower (p < 0.05) sulfate concentrations at the end of the incubation under aerobic (2.60 ± 0.09 mg/L) and anaerobic (0.36 ± 0.36 mg/L) conditions.

### Organic carbon content and composition

Prior to the incubations, the TOC concentration (all size fractions combined) was significantly higher for the SW-C soils (83.0 ± 24.0 mg/L) compared to FW-E (4.97 ± 0.82 mg/L) and FW-C soils (2.59 ± 0.63 mg/L; p < 0.05; Fig 5) despite the FW-E solid soils having a higher percent carbon content (Fig 4 in S1 Text). Prior to the incubations, TOC in the freshwater samples was dominated by the smallest NP+S size fraction with 64 ± 28% and 70 ± 10% of the aqueous TOC pool present as nanoparticles and soluble carbon for FW-C and FW-E, respectively (Fig 5). In contrast, only 23 ± 9% of the TOC was in the NP+S size fraction for the saline SW-C soils and most of the TOC (58 ± 9%) was in the largest particulate phase prior to incubation. Fine colloids were the smallest part of the aqueous TOC pool, representing 1 ± 2%, 12 ± 11%, and 19 ± 7% of TOC for FW-C, FW-E, and SW-C samples, respectively.

TOC concentrations increased on average for all size fractions, under both incubation conditions, and across all sites (p <0.05) with several exceptions—TOC for the FW-C soils did not vary significantly for the particulate fraction in the anaerobic incubation or the particulate and fine colloid fraction for the aerobic incubation, and likewise for FW-E soils did not vary significantly for the particulate and fine colloid fraction for the anaerobic incubation or particulate fraction for the aerobic incubation (p > 0.05). Across all sites, there was an average 2.8 and 1.8 times increase in particulate TOC, 12.3 and 5.9 times increase in fine colloidal TOC, and 7.2 and 3.6 times increase in nanoparticulate and soluble TOC under anaerobic and aerobic conditions, respectively. Thus, during the incubations, there was a shift towards a greater proportion of FC and NP+S TOC size fractions; this increase was most evident for the freshwater and saline wetland center sites.

At the end of the incubations, the fine colloidal fraction represented 44 ± 8% and 10 ± 17% of the TOC pool for FW-C, 6 ± 3% and 16 ± 4% of the TOC pool for FW-E, and 39 ± 5% and 34 ± 6% of the TOC pool for SW-C soils under anerobic and aerobic conditions, respectively. The nanoparticulate and soluble fraction represented 37 ± 12% and 82 ± 11% of the TOC pool for FW-C, 92 ± 2% and 73 ± 2% of the TOC pool for FW-E, and 30 ± 12% and 30 ± 2% of the TOC pool for SW-C soils under anerobic and aerobic conditions, respectively. Finally, the SW-C soils had substantially higher TOC concentrations compared to the other two soils under both anaerobic and aerobic conditions (Fig 5; p < 0.05).

**Fig 6:**
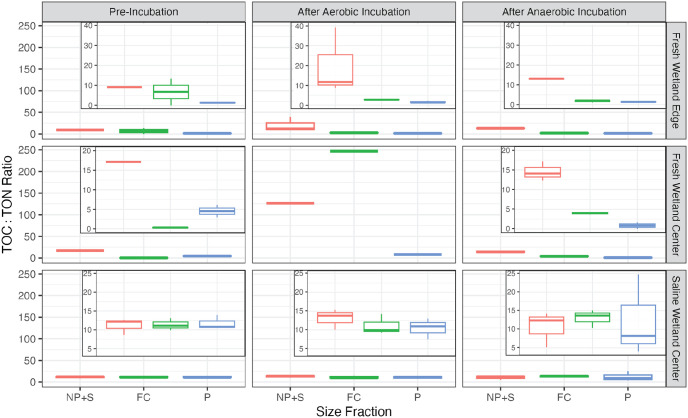
The ratio of total organic carbon to total dissolved nitrogen (C:N) across size fractions and incubation conditions. The smallest size fraction is nanoparticulates plus truly soluble material (NP+S; <0.1μm), followed by fine colloids (FC; 0.1–0.45μm), then particulates (P; 0.45–1μm). The insets in the left plot show a zoomed in y-axis.

For the saline soils, there was no significant difference in the TOC:TON ratio between size fractions or in the pre-incubated compared to incubated samples (p > 0.05; Fig 6). The average TOC:TON ratio for all size fractions combined was 11.4 ± 1.7 prior to incubation, 11.6 ± 2.6 under aerobic conditions and 12.0 ± 6.2 under anerobic conditions for SW-C soils (Fig 6). TOC:TON ratios became more variable across the different size fractions under anerobic conditions with average TOC:TON ratios of 12.3 ± 11.0, 13.0 ± 2.4, and 10.6 ± 4.8 for the P, FC, and NP+S size fractions, respectively for SW-C soils (Fig 6), but differences between size fractions were insignificant (p > 0.05).

Both freshwater soils had significantly lower TOC:TON ratios than the saline soils, with a pre-incubation average of 5.3 ± 5.3 and 5.4 ± 7.0 for FW-E and FW-C, respectively, when considering all size fractions (p < 0.05). Compared to the saline soils, there were also larger differences in TOC:TON ratios between size fractions for the FW-E soils, which ranged from 9.2 (n = 1), 6.7 ± 6.7, and 1.3 ± 0.3 for NP+S, FC, and P fractions, respectively, for the pre-incubation samples. Likewise, TOC:TON ratios for the FW-C soils ranged from 17.1 (n = 1), 0.3 ± 0.1, and 4.6 ± 2.2 for NP+S, FC, and P fractions, respectively, for the pre-incubation samples.

TOC:TON ratios in the smallest NP+S fraction increased for the FW-E soils after aerobic and anaerobic incubations (19.8 ± 16.8 and 13.1 ± 0.2, respectively), decreased for the FC size fraction (2.8 ± 0.2 and 1.8 ± 0.5, respectively), and remained relatively unchanged in the largest P fraction (1.6 ± 0.5 and 1.5 ± 0.5, respectively). For incubated FW-C soils, TOC:TON ratios in the NP+S fraction increased substantially under aerobic conditions (127, n = 1) and slightly decreased under anaerobic conditions (13.1 ± 2.7), increased for the FC fraction under aerobic and anaerobic conditions (247, n = 1 and 4.0 ± 0.3, respectively), increased for the P fraction under aerobic conditions (8.0, n = 1) and decreased for the P fraction under anerobic conditions (2.2 ± 2.4). Note that it was not possible to test the results presented in this paragraph for statistical significance because some replicates had TOC or TON concentrations of zero and incalculable ratios. In the solid soil phase, TOC:TON ratios decreased under both incubation conditions for SW-C, increased under both incubation conditions for FW-C, and increased under aerobic conditions but decreased under aerobic conditions for FW-E (Fig 6 in S1 Text).

In terms of molecular OM characterization via FT-ICR-MS, we did not detect significant differences between any of the size fraction (Tables 1–3 in S1 Text), so we focus our discussion on a summary of the average composition across all size fractions (Table 1). The lack of variability across size fractions is likely because all samples needed to be subjected to solid phase extraction to prepare samples for analysis; the extraction process likely biases the results towards the composition of soluble TOC. For both freshwater soils, there was a slight increase in the number of unique features (i.e., number of peaks) after being incubated in aerobic conditions, but this change was not significant (p > 0.05). Freshwater soils, however, did exhibit a significant decrease in the number of unique features under anaerobic conditions (p < 0.05). In contrast there was a significant decrease in features in the saline soil under both incubation conditions (Table 1; p < 0.05) demonstrating a loss of diversity for saline soils regardless of redox state.

**Table 1:** Summary of the total number of peaks detected via FT-ICR-MS and the calculated percent contribution of different compound classes to the portion of the TOC pool captured within the analytical window. The average of all size fractions (<1 μm, <0.45 μm, and <0.1 μm) is presented here, and a breakdown by size fraction can be found in Tables 1–3 in S1 Text.

Soil and Incubation Type	FW-E Pre-Incubation	FW-E Aerobic Incubation	FW-E Anaerobic Incubation	FW-C Pre-Incubation	FW-C Aerobic Incubation	FW-C Anaerobic Incubation	SW-C Pre-Incubation	SW-C Aerobic Incubation	SW-C Anaerobic Incubation
**Compound Class**									
Amino Sugars	5 ± 1	3 ± 0	2 ± 0	5 ± 1	3 ± 1	2 ± 1	4 ± 1	4 ± 1	4 ± 1
Carbohydrates	4 ± 2	2 ± 0	1 ± 1	3 ± 2	2 ± 1	1 ± 0	3 ± 1	2 ± 0	2 ± 0
Condensed Hydrocarbons	12 ± 5	18 ± 4	22 ± 3	14 ± 4	19 ± 3	22 ± 3	14 ± 6	12 ± 5	13 ± 5
Lignin	38 ± 3	50 ± 5	45 ± 1	39 ± 4	48 ± 2	45 ± 2	40 ± 4	35 ± 4	32 ± 4
Lipids	9 ± 3	4 ± 1	6 ± 2	8 ± 3	4 ± 1	6 ± 2	9 ± 5	15 ± 5	17 ± 5
Other	1 ± 0	0 ± 0	0 ± 0	1 ± 0	0 ± 0	0 ± 0	1 ± 0	0 ± 0	0 ± 0
Proteins	22 ± 5	12 ± 1	11 ± 3	21 ± 6	11 ± 2	11 ± 3	17 ± 5	21 ± 5	22 ± 4
Tannins	7 ± 3	10 ± 2	12 ± 2	8 ± 3	11 ± 2	12 ± 2	10 ± 3	8 ± 2	8 ± 2
Unsaturated Hydrocarbons	1 ± 1	1 ± 0	0 ± 0	1 ± 1	1 ± 0	1 ± 0	1 ± 1	2 ± 1	2 ± 1
Number of Peaks	9,888 ± 2,076	10,540 ± 1,673	8,773 ± 1,572	9,706 ± 2,136	10,390 ± 2,432	8,802 ± 1,513	9,688 ± 754	7,976 ± 911	7,243 ± 862

Lignin-like molecules were the dominant compound class for all soils both pre- and post-incubation under both conditions, contributing from 29–49% of the aqueous TOC pool. The proportion of lignin-like TOC in the saline soils significantly decreased under both incubation conditions in contrast to an increase in the proportion of lignin-like TOC for both freshwater soils under both incubation conditions (Table 1; p < 0.05). Protein-like molecules were the second most abundant compound class, and similarly had divergent behavior between freshwater and saline soils. In contrast to lignin-like molecules, the proportional abundance of protein-like molecules decreased for both freshwater soils and increased in the saline soils following both incubation conditions (p < 0.05). The third most abundant compound classes were lipid-like and condensed hydrocarbon molecules. Condensed hydrocarbons increased in proportional abundance following both incubation conditions in the freshwater soils (p < 0.05) but did not change substantially in the saline soils. The relatively labile amino sugar-like TOC fraction only made up ~5% of the TOC pool prior to incubation for all soils but decreased significantly for both freshwater soils under both incubation conditions (p < 0.05) but remained unchanged in the incubated saline soils (p > 0.05). Carbohydrate-like TOC was similarly abundant prior to incubation and decreased in all soils under all incubation conditions (p < 0.05). Tannin-like TOC followed a similar trend, decreasing for all soils under all incubation conditions (p < 0.05). Unsaturated hydrocarbons made up a minimal fraction of the TOC pool (i.e., 0–3%) across the soil types and incubation conditions (Table 1).

To compare molecular level data with bulk analyses, we also computed C:N ratios derived from FT-ICR-MS data. Interestingly, the C:N ratios calculated with FT-ICR-MS data were substantially higher compared to bulk C:N ratios with pre-incubation averages of 30.5 **±** 4.0, 32.0 **±** 5.2, and 31.9 **±** 1.7 for FW-E, FW-C, and SW-C soils, respectively. FT-ICR-MS-derived C:N ratios significantly decreased (p < 0.05) for both freshwater soils under aerobic conditions (28.8 **±** 2.7) and increased (p < 0.05) for SW-C soils under both aerobic (36.4 **±** 3.8) and anaerobic conditions (40.0 **±** 4.5), which in both cases contrasts the trends observed in bulk C:N ratios.

### Greenhouse gas production

After 21 days under aerobic conditions, each soil type produced significantly different amounts of CO_2_ compared to one another (p < 0.05) with SW-C soils producing the least (7.72 ± 0.4 mmol C / kg dry soil), followed by FW-C (9.17 ± 0.82 mmol C / kg dry soil), and FW-E (13.8 ± 2.14 mmol C / kg dry soil). In addition to the FW-E soils producing more CO_2_ under aerobic conditions, the rate of CO_2_ production peaked after seven days (Fig 1), resulting in a less linear behavior over the course of the 21-day incubation (R^2^ = 0.72) compared to FW-C (R^2^ = 0.94) and SW-C soils (R^2^ = 0.98).

**Fig 7:**
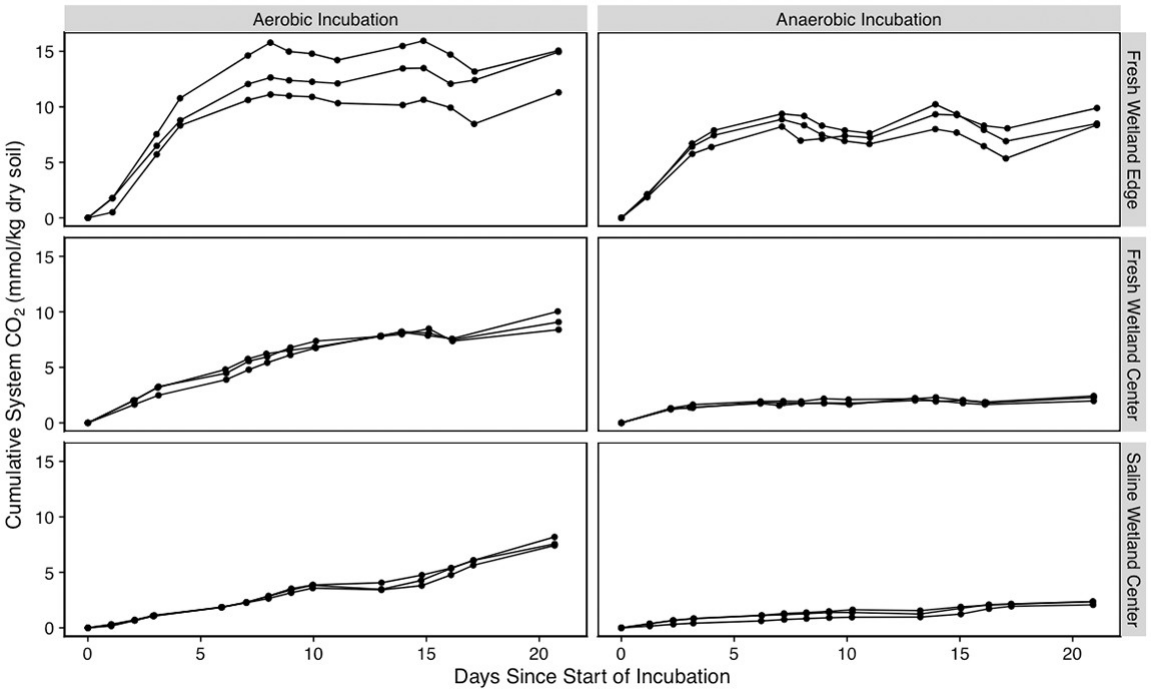
Carbon dioxide production. Net carbon dioxide accumulation during aerobic (left) and anaerobic (right) incubations expressed per kg of dry soil.

For all three types of soils, total CO_2_ production over the 21-day experiment was lower under anaerobic conditions compared to the same soil incubated under aerobic conditions. Aerobic CO_2_ production was 4.1, 1.5, and 3.4 times greater than anaerobic production for FW-C, FW-E, and SW-C soils, respectively (p < 0.05). Likewise, when considering all soil types together, the aerobic experiments produced 2.3 times more CO_2_ than the anaerobic experiments at the end of the experiment (p < 0.05). After 21 days under anaerobic conditions, the FW-C and SW-C soils produced similar amounts of CO_2_ (2.22 ± 0.23 and 2.27 ± 0.17 mmol C / kg dry soil, respectively; p > 0.05), whereas the FW-E soils produced significantly more CO_2_ compared to the other soil types (8.92 ± 0.85 mmol C / kg dry soil; p < 0.05). The temporal behavior of CO_2_ production observed in the anaerobic experiments (Fig 7) were similar to the aerobic experiments with FW-E peaking around day 4 and exhibiting the least linear behavior (R^2^ = 0.66) compared to FW-C (R^2^ = 0.73) and SW-C (R^2^ = 0.93).

**Fig 8:**
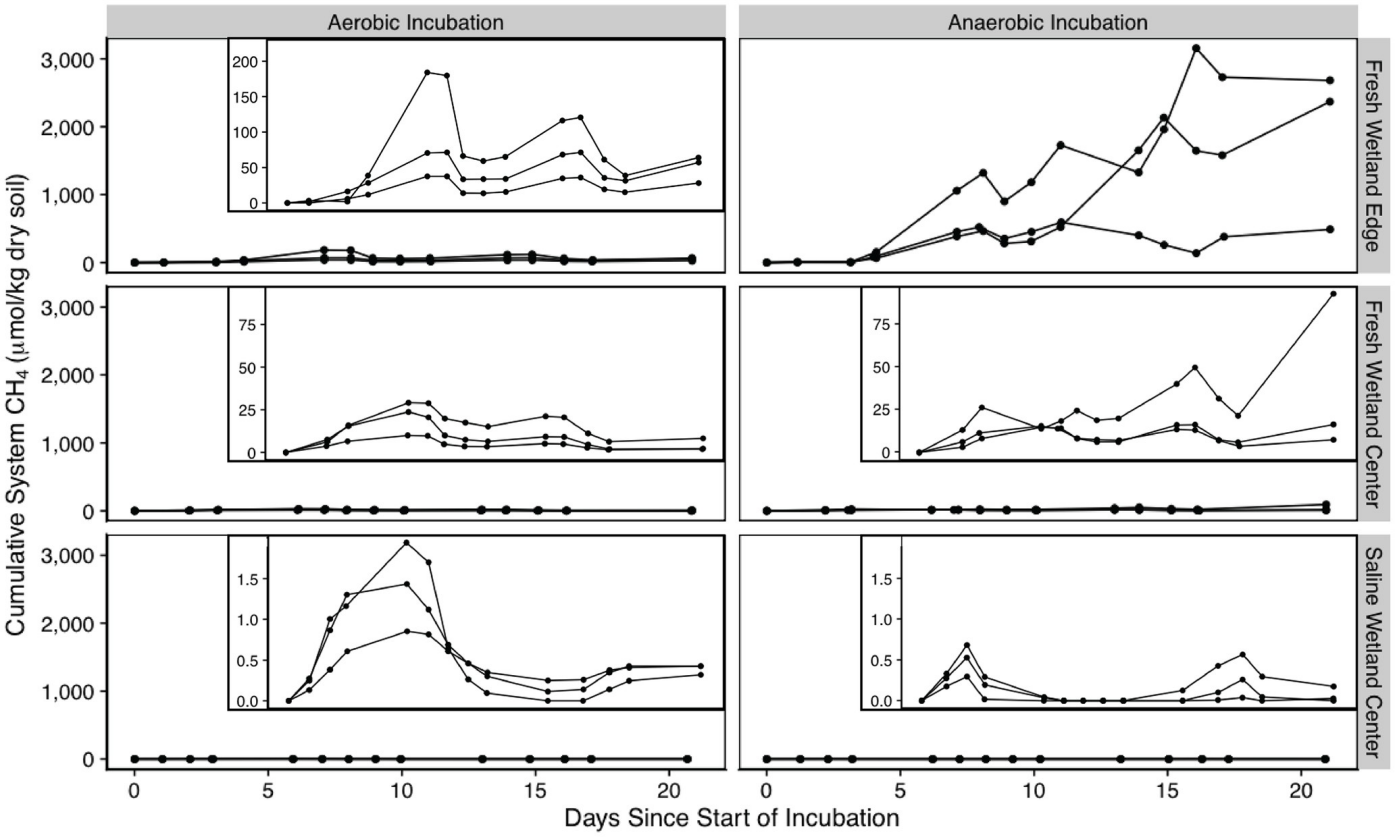
Methane production. Net methane accumulation during incubations aerobic (left) and anaerobic (right) expressed per kg of dry soil. Insets show a zoomed in y-axis.

A negligible amount of CH_4_ production was detected for the saline and freshwater wetland center soils under aerobic conditions, whereas and appreciable amount of methane was produced in the FW-E soils (Fig 8). After 21 days under aerobic conditions, SW-C soils produced the least CH_4_ (0.390 ± 0.061 μmol C / kg dry soil), followed by FW-C (4.16 ± 3.56 μmol C / kg dry soil), and FW-E (49.7 ± 47.0 μmol C / kg dry soil). Similar to CO_2_ production, the difference in aerobic methane production was significant between each soil type (p > 0.05).

The minimal methane production under aerobic conditions was to be expected; however, interestingly, even under anaerobic conditions CH_4_ production was low for FW-C and SW-C soils [38.9 ± 47.0 and 0.067 ± 0.094 μmol C / kg dry soil by the end of the incubation, respectively; (Fig 8)]. FW-E soils produced more methane under aerobic conditions than the other two soils did under anaerobic conditions. FW-E soils also had substantially higher amounts of CH_4_ (p < 0.05) produced after 21 days under anaerobic conditions (1,845 ± 1,186 μmol C / kg dry soil) with substantial methane production not initiating until day four; this lag was also observed in the aerobic FW-E incubations. The difference in anaerobic methane production was significant between each soil type (p < 0.05).

The molar ratio of CO_2_:CH_4_ varied substantially between incubation conditions, ranging from a minimum value of 2.51 in the FW-E anaerobic experiment to a maximum value of 225,206 in the SW-C anaerobic experiment (Fig 7 in S1 Text). The average CO_2_:CH_4_ ratio for the final time point (day 21) of the anaerobic experiments was lowest for FW-E (8.98 **±** 9.76), followed by FW-C (152 **±** 144) and SW-C (49,295 **±** 52,940); despite the large differences in mean ratios at the end of the experiment, differences between soil types were not significant given the large variability between triplicates (p > 0.05) except for the difference between FW-E and FW-C soils, which was significant (p < 0.05). The average CO_2_:CH_4_ ratio for the final time point (day 21) of the aerobic experiments was similarly lowest in the FW-E soils (299 **±** 89.5), followed by FW-C (3,386 **±** 2,134) and SW-C (20,121 **±** 3,186); in this case the difference between each soil type was a significant (p < 0.05).

## Discussion

### Physiochemical conditions of the incubations

The increase in ORP after aerobic (oxidizing) incubation and a decrease in anaerobic (reducing) conditions observed in both freshwater soils was expected (Fig 1 in S1 Text). However, the counter-intuitive decrease in ORP in the saline soil even under aerobic conditions may be related to the association of increased ionic concentrations in saline soils with decreased oxygen solubility, which leads to lower redox potentials [32]. Porewater dissolved oxygen at the time of soil collection was only 2.2 mg/L at the saline site and all soils were conditioned in an anaerobic chamber for 4 days prior to incubation, so the introduction of oxygen was predicted to increase ORP due to the high redox potential of oxygenated water as a redox pair [57]. Additionally, anaerobic saline soil incubations were the only ones to reach negative ORP values. The increase in alkalinity and pH after anaerobic incubation of soil-water solutions is also consistent with previous incubation studies [19, 58]. Increases in pH in soils subjected to anoxia have been attributed to dissolution of metal oxyhydroxides and oxides [59, 60] and to consumption of H^+^ in Fe-oxide reduction [61, 62]. In relation to carbon mobility, Grybos et al. [63] observed increased DOC release under reducing conditions due to associated pH increases and reduction of reactive iron and manganese, which aligns with our observation of elevated DOC levels in the anaerobic incubations (Fig 5).

Alkalinity increased to the greatest degree in saline soils in which sulfate reduction likely occurred, a reaction type that yields bicarbonate [64]. The substantial increase in alkalinity for all soils under anaerobic conditions suggests that the affinity for coastal wetland ecosystems to export alkalinity to the ocean [3] is strongly linked to their typically anoxic condition [65].

In addition to the observed physicochemical changes over the course of the incubations, there was also variability in ORP, alkalinity and pH across size fractions (Figs 1–3 in S1 Text). The observation of higher pH in the <0.1 μm fraction compared to <0.45 μm, <1 μm, and unfiltered samples perhaps indicates an abundance of high molecular weight organic acids (humic acids) in the larger fractions and/or metal complexations of humic or fulvic acids [66]. ORP was similarly highest in the <0.1 μm fraction for the saline soil both before and after incubation. This is likely due to the presence of more reduced analytes in the larger fractions (e.g., Fe^2+^ complexations; see section 4.3) compared to more oxidized organic material in the <0.1 μm fraction [67].

### Organic carbon content and composition

Substantial amounts of carbon were released from the soil and accumulated in the aqueous solution over the course of the incubations (Fig 7). As in prior studies [68–70], TOC concentrations at the end of the incubation were far greater under anaerobic conditions compared to aerobic (Fig 5). Anaerobic water saturated soils tend to exhibit higher concentrations of organic matter *in situ* than aerobic soils due to multiple physical, chemical, and metabolic factors [71]. For example, nitrate-reducing conditions have been shown to promote DOC release while sulfate-reducing or methanic conditions limit DOC release [72]. Increased salinity is also thought to induce flocculation of OM, particularly humic substances, and inhibit extraction of OM from soils [73]. However, this contrasts our finding that the greatest DOC concentrations by far were observed in our saline wetland soils. Based on the differences we observed between the same soils exposed to aerobic or anaerobic conditions, some potential mechanisms limiting carbon biodegradation and promoting aqueous phase carbon accumulation in these experiments may be the low energetic efficiency of anaerobic decomposition [60] and anoxic constraint of phenol oxidase enzymes [74].

Final concentrations of TOC in the saline soil incubates were an order of magnitude higher than in freshwater soils, which is interesting given that salinity is associated with increased activity of carbon-degrading enzymes [75] and thus could be expected to have increased biodegradation. Likewise, soil extractions with seawater tend to yield lower DOC release, potentially attributable in part due to flocculation of OM in response to higher ionic strength [73, 76]. However, in the case of our incubations, the saline soils were exposed to freshwater. Decreasing the ionic strength of soil-water solutions can expand the diffuse double layer, causing disaggregation and destabilization of associations between OM and minerals and an increase in released TOC [77, 78].

Iron cycling is another factor that may have influenced the amount and size of TOC released for the different soils under anerobic conditions. Mineral iron oxides bound to OM can be reduced under anoxic conditions, releasing the OM formerly bound in soil [63, 67]. Along these lines, Wang et al. [79] implicated iron oxidation in regulating phenol oxidation activity in oxic conditions and counteracting the “latching” effect of oxygen to phenol oxidases. Under oxic conditions, iron (hydr)oxides generally contribute to OM storage [80] while in anoxic conditions iron hydroxides acting as terminal electron acceptors can increase OM decomposition [81, 82]. Indeed, we observed a large increase in all size fractions of TOC for both the SW-C and FW-C soils incubated in anerobic conditions, which had high levels of reduced Fe^2+^. On the other hand, the FW-E soil showed no evidence of iron reduction and did not see an increase in fine colloidal or particulate TOC (Figs 5 and 7). The majority of OC of the size fractions examined was present in the nanoparticulate and truly soluble fraction in freshwater soils, which follows the trend seen by Afsar et al. [19] in experiments with another freshwater wetland soil. Their observation of OC in nanoparticulate size fractions being more redox-dependent than in particulate size fractions also agrees with the higher increases in TOC concentrations observed here in the smallest size fraction. Two exceptions to this trend were anaerobic incubations of freshwater and saline wetland center soils; these soils also showed low CO_2_ production compared to aerobic samples.

One other factor that may have resulted in the observed differences in TOC size fractions between the freshwater and saline sites is different vegetation characteristics of the sites. The saline Beaver Creek soils had a high abundance of root biomass and other plant detritus associated with marsh grass [35], which likely contributed to the high particulate TOC abundance compared to the freshwater soils. Vegetation composition at the freshwater site, Old Woman Creek, on the other hand is more variable over time due to complex flooding and drying regimes; depending on annual water levels, vegetation at the wetland site that was sampled can vary between different macrophytes such as water lily, lotus, or cattail cover [83]. At the time of sampling, the freshwater wetland sites were not inundated and had minimal live vegetation present.

Evaluating the TOC pool by size fractions revealed large differences in bulk composition between sites and size fractions. The freshwater soils not only had a higher proportion of truly soluble and nanoparticulate TOC that remained unassociated with minerals, but they also had a substantially lower average TOC:TON ratio than the saline soils (Fig 6). The low average pre-incubation ratios of 5.3 **±** 5.3 and 5.4 **±** 7.0 for FW-E and FW-C, respectively, are reflective of microbial detritus, whereas the C:N ratios for SW-C of 11.4 **±** 1.7 are more reflective of vegetation detritus [84]. Microbially-derived OM with low C:N ratios in soils is often considered highly bioavailable in soils [85]. Though interestingly, the TOC:TON ratio of the truly soluble and nanoparticulate fraction for the freshwater soils was closer to values expected for vegetation detritus suggesting that perhaps the byproducts of microbial degradation and/or dead microbial biomass were the primary OM types that formed the colloidal complexes observed in the freshwater soils.

Finally, it is worth noting that the observed differences between molecular level and bulk OM characterizations highlight the fact that extracting samples and analyzing via mass spectrometry substantially narrows the analytical window and range of molecules considered for calculations such as C:N ratios [86]. The observed differences in bulk OM composition, which provided evidence for varying bioavailability between soil types and redox treatments, would not have been as evident without examining the elemental composition of different size fractions. Our characterization of OM composition via FT-ICR-MS, alone, would have suggested a more homogenous composition across soils and incubations (Table 1) due to the smaller analytical window this method provides.

### Greenhouse gas production and competing redox reactions

Constraining the drivers of coastal ecosystem GHG fluxes remains a challenge given the highly variable responses in GHG cycling to factors such as salinity exposure and hydrological variability. For example, observed responses of soils to increasing salinity include stimulation of OM decomposition and CO2 fluxes [87], inhibition of decomposition [88–90], stimulation of CH_4_ release with inhibition of CO_2_ [90], negligible methane emissions in polyhaline marshes [5], and a quadratic relationship with initial decrease in decomposition with increasing salinity followed by heightened decomposition at higher salinities [91].

The low methane production in both fresh and saline wetland center soils we observed is likely due to the reduction of species (e.g., NO_3_^-^, MnO_2_, Fe^3+^, SO_4_^2-^) higher on the redox ladder outcompeting methanogenesis. Nitrate was depleted for all soil types under anerobic conditions suggesting that nitrate reduction likely did not prevent methanogenesis from occurring for any of the soils. Iron reduction was prominent for both saline and freshwater wetland center soils as shown by high post-incubation Fe^2+^ concentrations (Fig 3) and was likely an important reaction competing with methanogenesis similar to findings from other experimental studies [31]. Interestingly, the FW-E soils, which produced 47 times more methane under anaerobic conditions than the FW-C soils (Fig 8), had higher pre-incubation concentrations of total iron than the FW-C soil (Fig 2). However, FW-E did not produce appreciable amounts of Fe^2+^, suggesting that significant iron reduction did not occur in the wetland edge soil and/or there was not a prominent solid phase iron source. The FW-E soils exhibited optimal conditions for anaerobic methanogenesis and were apparently also suitable for producing methane under aerobic conditions [26].

A variety of factors are known to influence the availability of Fe^3+^ in soils, including acidic pH and low ORP [92]. The freshwater wetland center soil, which appeared to host iron reduction, had lower pH values during anaerobic incubations than the wetland edge soil, though not as acidic as conditions typically associated with high iron solubility [93]. Low redox potential is also associated with iron reduction, and the FW-E soil, which had no increase in Fe^2+^, did have higher redox potentials than the other soils. Methane production took about four days to initiate in the FW-E soils, perhaps because of competing redox reactions that exhausted all available terminal electron acceptors (Fig 8). This difference in iron cycling between the two freshwater sites is perhaps related to the fact that FW-C soils are more frequently flooded and anoxic compared to FW-E, which is only periodically inundated [41].

Sulfate reduction was another important competing redox reaction in the saline soil under anaerobic conditions. Although we cannot discount sulfate reduction as a competing reaction with methanogenesis for the freshwater wetland center soil, which had high levels of sulfate (Fig 4), the odor of H_2_S emerging from the saline samples is clear evidence that sulfate reduction was active for the saline soils. Solid phase sulfur content was relatively high in the saline soil prior to incubations and decreased markedly under both incubation conditions (Fig 5 in S1 Text). Potential sources of sulfate might include oxidation of iron and sulfur containing minerals under aerobic conditions or reductive dissolution of minerals under anaerobic conditions [94]. It is unclear how long the soils would need to be incubated (or flooded in the case of the natural ecosystem setting) to exhaust the large source of mineral sulfur and initiate methanogenesis, particularly for the saline soil.

Experimental results from this study highlight the importance in considering the diversity of size fractions present in soil-water matrices for interpreting drivers of GHG production. For example, high Fe^2+^ and low nitrate levels in the two soils with low anaerobic methane production suggest that iron reduction played at least some role in limiting methanogenesis. However, measuring dissolved iron via filtration would have masked this finding. The majority of Fe^2+^ in soils with high concentrations was measured in the largest particulate size fraction (0.45–1 μm), and elevated Fe^2+^ would not have been detected if we focused solely on dissolved phases that passed through a typical 0.45 μm filter (Fig 3). This is counterintuitive considering Fe^2+^ is the soluble form of iron. We hypothesize that this high Fe^2+^ content in the particulate phase is related to complexation of Fe^2+^ with organic matter >0.45 μm in size) similar to recent evidence of synthesis of stable Fe^2+^-OM complexes [95]. It is also possible that particulate secondary Fe^2+^/Fe^3+^ minerals with or without sorbed or structural Fe^2+^ could be present considering that Fe^2+^ containing ferrihydrite can be an intermediate product of ferrihydrite transformation to green rust [96].

Colloids greater than 0.45 μm are also often excluded from studies of OM characterization and/or GHG production, but in this study the larger size fraction was key in providing evidence for iron reduction as a potential mechanism for mineral-associated TOC release. Considering colloidal and particulate size fractions of TOC also yielded insight into the mechanisms underlying different responses in aerobic CO_2_ production across the different soil types. TOC quantity was clearly not the primary driver of aerobic respiration in our experiments considering the low CO_2_ production for SW-C soils (Fig 7), which had an order of magnitude higher TOC content (Fig 5). Rather, carbon quality and/or size fractionation was likely a key constraint on aerobic respiration. Aerobic CO_2_ production was highest for both freshwater soils, which had a much higher proportion of pre-incubation OM in the NP+S fraction (64 **±** 28% and 70 **±** 10% for FW-C and FW-E, respectively) compared to the SW-C soil (24 **±** 9%). In the case of FW-E soils, aerobic CO_2_ production plateaued, suggesting that the system became labile carbon limited as the NP+S fraction got replaced by larger colloidal and particulate carbon. These findings contrast conceptual models for OM bioavailability in aquatic settings. For example, larger colloidal and particulate OM is thought to be more bioavailable in marine surface waters (Benner and Amon, 2015), but active association or disassociation with mineral surfaces may complicate this model in wetland systems [37].

## Conclusions

The balance between coastal wetland carbon storage, lateral export, and decomposition is susceptible to hydrological changes that both marine and inland coasts (i.e., large lakes) will experience in the coming decades including relative sea level rise, lake level variability along large freshwater coastlines, and increasing occurrence and severity of extreme weather events [97–100]. These hydrological disturbances are expected to have varying impacts on biogeochemical processes depending on differences in factors such as salinity exposure, flooding frequency, terminal electron acceptor abundance, and redox state variability [56, 101–105]. Therefore, an understanding of the mechanisms underlying the compounding effects of these disturbances on wetland soil OM transport, decomposition, and storage is particularly important for both predicting and managing the future state of coastal carbon cycling [106–108].

In this study, we experimentally probed several major gaps in our understanding of the properties and processes that mediate GHG emissions from coastal wetlands and the mobility of carbon in coastal wetland soils across a more complete size class spectrum than is typically studied. By characterizing a spectrum of different size fractions (e.g., soluble, nanoparticulates, fine colloids, and particulates), we were able to develop mechanistic insight into why and under what conditions certain redox reactions can limit methanogenesis. We found that reduction of mineral-phase iron was one major factor limiting methanogenesis in both fresh and saline wetland center soils along with sulfate reduction in the saline soil. Surprisingly, Fe^2+^ did not accumulate in the truly soluble size fraction, but instead was present primarily in larger size fractions as either Fe^2+^-OM complexes or Fe^2+^ containing ferrihydrite, which highlights the need to evaluate the presence of presumably soluble materials that might associate with colloids under certain conditions. Measuring only truly dissolved iron would have masked this important finding and limited our ability to understand why methanogenesis was inhibited in a freshwater wetland.

We used the same size fractionation technique to characterize the aqueous organic matter pool to mechanistically understand why aerobic CO_2_ production varied substantially across the three different studied soils. We found that soils with a higher proportion of soluble molecules had substantially higher rates of aerobic respiration compared to soils with a higher proportion of colloidal and particulate carbon. Likewise, we found that carbon quality was perhaps more important than carbon quantity in driving high respiration rates. In this case, characterizing DOC as a function of size class substantially improved our ability to interpret the mechanisms underlying changes in CO_2_ production.

Finally, we found that colloidal organic matter could be a prominent, and overlooked, component of the pool of carbon that is mobile in wetland soil environments, particularly for the saline soils we investigated. The high proportion of colloidal OM found in the saline wetland soil, OM that typically goes uncharacterized, may be a major component of the carbon that is laterally transported from coastal wetlands to the ocean. The release of this OM is likely linked to cycling of redox sensitive elements such as iron and sulfur. Depending on whether colloidal OM released from coastal wetlands persists or is remineralized in open water environments, this material export can result in either an additional major carbon sink or source of atmospheric carbon from coastal systems, respectively. Collectively, our results highlight the value of continuing to expand the analytical window that we examine Earth’s carbon cycle through. While colloidal size characteristics may seem like a subtle geochemical nuance, such fine scale details may influence global scale carbon balances.

## Supporting information

S1 Text Supporting figures and tables. The file “S1 Text.pdf” contains additional data visualizations and summaries.
(PDF)

## References

[1] Spivak AC, Sanderman J, Bowen JL, Canuel EA, Hopkinson CS. Global-change controls on soil-carbon accumulation and loss in coastal vegetated ecosystems. Nat Geosci. 2019;12(9):685–92. doi: 10.1038/s41561-019-0435-2

[2] McLeod E, Chmura GL, Bouillon S, Salm R, Björk M, Duarte CM, et al. A blueprint for blue carbon: toward an improved understanding of the role of vegetated coastal habitats in sequestering CO2. Front Ecol Environ. 2011;9(10):552–60.

[3] Santos IR, Burdige DJ, Jennerjahn TC, Bouillon S, Cabral A, Serrano O, et al. The renaissance of Odum’s outwelling hypothesis in ’Blue Carbon’ science. Estuar Coast. 2021:107361. doi: 10.1016/j.ecss.2021.107361

[4] Marín-Spiotta E, Gruley K, Crawford J, Atkinson E, Miesel J, Greene S, et al. Paradigm shifts in soil organic matter research affect interpretations of aquatic carbon cycling: transcending disciplinary and ecosystem boundaries. Biogeochemistry. 2014;117:279–97.

[5] Poffenbarger HJ, Needelman BA, Megonigal JP. Salinity influence on methane emissions from tidal marshes. Wetlands. 2011;31(5):831–42.

[6] Diefenderfer HL, Cullinan VI, Borde AB, Gunn CM, Thom RM. High-frequency greenhouse gas flux measurement system detects winter storm surge effects on salt marsh. Glob Chang Biol. 2018;24(12):5961–71. doi: 10.1111/gcb.14430 30152082

[7] Ward ND, Megonigal JP, Bond-Lamberty B, Bailey VL, Butman D, Canuel EA, et al. Representing the function and sensitivity of coastal interfaces in Earth system models. Nat Commun. 2020;11(1):2458. doi: 10.1038/s41467-020-16236-2 32424260 PMC7235091

[8] Sulman BN, Wang J, LaFond-Hudson S, O’Meara TA, Yuan F, Molins S, et al. Integrating tide‐driven wetland soil redox and biogeochemical interactions into a land surface model. J Adv Model Earth Syst. 2024;16(4). doi: 10.1029/2023ms004002

[9] Rodrigo-Comino J, López-Vicente M, Kumar V, Rodríguez-Seijo A, Valkó O, Rojas C, et al. Soil science challenges in a new era: a transdisciplinary overview of relevant topics. Air, Soil and Water Research. 2020;13:1178622120977491.

[10] Dong X, Cohen MJ, Martin JB, McLaughlin DL, Murray AB, Ward ND, et al. Ecohydrologic processes and soil thickness feedbacks control limestone-weathering rates in a karst landscape. Chem Geol. 2018. doi: 10.1016/j.chemgeo.2018.05.021

[11] Grabs T, Bishop K, Laudon H, Lyon SW, Seibert J. Riparian zone hydrology and soil water total organic carbon (TOC): implications for spatial variability and upscaling of lateral riparian TOC exports. Biogeosciences. 2012;9(10):3901–16.

[12] Talbot CJ, Bolster D, Medvigy D, Jones SE. A Terrestrial‐Aquatic Model Reveals Cross‐Scale Interactions Regulate Lateral Dissolved Organic Carbon Transport From Terrestrial Ecosystems. J Geophys Res Biogeosci. 2022;127(5):e2021JG006604.

[13] Menendez A, Tzortziou M, Neale P, Megonigal P, Powers L, Schmitt‐Kopplin P, et al. Strong dynamics in tidal marsh DOC export in response to natural cycles and episodic events from continuous monitoring. J Geophys Res Biogeosci. 2022;127 (7):e2022JG006863.

[14] Xiao K, Zhang P, Santos IR, Wang JJ, Li Z, Wang X, et al. Tidal pumping controls dissolved organic matter properties and outwelling from mangrove groundwater to coastal water. Water resources research. 2023;59(3):e2022WR033913.

[15] Tzortziou M, Neale PJ, Osburn CL, Megonigal JP, Maie N, JaffÉ R. Tidal marshes as a source of optically and chemically distinctive colored dissolved organic matter in the Chesapeake Bay. Limnol Oceanogr. 2008;53(1):148–59. doi: 10.4319/lo.2008.53.1.0148

[16] Bogard MJ, Bergamaschi B, Butman D, Anderson F, Knox S, Windham‐Myers L. Hydrologic export is a major component of coastal wetland carbon budgets. Global Biogeochem Cycles. 2020. doi: 10.1029/2019GB006430

[17] Knoke M, Dittmar T, Zielinski O, Kida M, Asp NE, de Rezende CE, et al. Outwelling of reduced porewater drives the biogeochemistry of dissolved organic matter and trace metals in a major mangrove‐fringed estuary in Amazonia. Limnology and Oceanography. 2024;69(2):262–78.

[18] Fettrow S, Jeppi V, Wozniak A, Vargas R, Michael H, Seyfferth AL. Physiochemical Controls on the Horizontal Exchange of Blue Carbon Across the Salt Marsh‐Tidal Channel Interface. J Geophys Res Biogeosci. 2023;128(6):e2023JG007404.

[19] Afsar MZ, Goodwin C, Beebe TP Jr, Jaisi DP, Jin Y. Quantification and molecular characterization of organo-mineral associations as influenced by redox oscillations. Sci Total Environ. 2020;704:135454. doi: 10.1016/j.scitotenv.2019.135454 31837876

[20] Maher DT, Santos IR, Golsby-Smith L, Gleeson J, Eyre BD. Groundwater‐derived dissolved inorganic and organic carbon exports from a mangrove tidal creek: The missing mangrove carbon sink? Limnology and Oceanography. 2013;58(2):475–88.

[21] Ho DT, Ferrón S, Engel VC, Anderson WT, Swart PK, Price RM, et al. Dissolved carbon biogeochemistry and export in mangrove-dominated rivers of the Florida Everglades. 2017.

[22] Chu SN, Wang ZA, Gonneea ME, Kroeger KD, Ganju NK. Deciphering the dynamics of inorganic carbon export from intertidal salt marshes using high-frequency measurements. Mar Chem. 2018;206:7–18. doi: 10.1016/j.marchem.2018.08.005

[23] Schmidt MWI, Torn MS, Abiven S, Dittmar T, Guggenberger G, Janssens IA, et al. Persistence of soil organic matter as an ecosystem property. Nature. 2011;478(7367):49–56. doi: 10.1038/nature10386 21979045

[24] Patel KF, Bond-Lamberty B, Jian J, Morris KA, McKever SA, Norris CG, et al. Carbon flux estimates are sensitive to data source: a comparison of field and lab temperature sensitivity data. Environmental Research Letters. 2022;17(11):113003.

[25] Freeman C, Ostle N, Kang H. An enzymic’latch’on a global carbon store. Nature. 2001;409(6817):149-. doi: 10.1038/35051650 11196627

[26] Keppler F, Boros M, Frankenberg C, Lelieveld J, McLeod A, Pirttilä AM, et al. Methane formation in aerobic environments. Environmental Chemistry. 2009;6(6):459–65.

[27] Vincent SGT, Jennerjahn T, Ramasamy K. Environmental variables and factors regulating microbial structure and functions. Microbial communities in coastal sediments. 2021:79–117.

[28] Machado-Silva F, Weintraub MN, Ward ND, Doro KO, Regier PJ, Ehosioke S, et al. Short-Term Groundwater Level Fluctuations Drive Subsurface Redox Variability. Environ Sci Technol. 2024;58(33):14687–97. doi: 10.1021/acs.est.4c01115 39115966

[29] Weston NB, Vile MA, Neubauer SC, Velinsky DJ. Accelerated microbial organic matter mineralization following salt-water intrusion into tidal freshwater marsh soils. Biogeochemistry. 2011;102(1):135–51. doi: 10.1007/s10533-010-9427-4

[30] Feng J, Hsieh YP. Sulfate reduction in freshwater wetland soils and the effects of sulfate and substrate loading. Wiley Online Library, 1998 0047–2425.

[31] Helton AM, Ardón M, Bernhardt ES. Hydrologic Context Alters Greenhouse Gas Feedbacks of Coastal Wetland Salinization. Ecosystems. 2019;22(5):1108–25. doi: 10.1007/s10021-018-0325-2

[32] Herbert ER, Boon P, Burgin AJ, Neubauer SC, Franklin RB, Ardón M, et al. A global perspective on wetland salinization: ecological consequences of a growing threat to freshwater wetlands. Ecosphere. 2015;6(10):1–43.

[33] Parkin TB. Soil microsites as a source of denitrification variability. Soil Science Society of America Journal. 1987;51(5):1194–9.

[34] Bailey VL, Smith AP, Tfaily M, Fansler SJ, Bond-Lamberty B. Differences in soluble organic carbon chemistry in pore waters sampled from different pore size domains. Soil Biol Biochem. 2017;107:133–43.

[35] Sengupta A, Indivero J, Gunn C, Tfaily MM, Chu RK, Toyoda J, et al. Spatial gradients in soil-carbon character of a coastal forested floodplain are associated with abiotic features, but not microbial communities. Biogeosciences. 2019;16(19):3911–28. doi: 10.5194/bg-2019-193

[36] Deb S, Mandal B. Soils and sediments of coastal ecology: A global carbon sink. Ocean & Coastal Management. 2021;214:105937.

[37] Yan J, Manelski R, Vasilas B, Jin Y. Mobile colloidal organic carbon: an underestimated carbon pool in global carbon cycles? Frontiers in Environmental Science. 2018;6:148.

[38] Afsar MZ, Vasilas B, Jin Y. Organo-mineral associations and size-fractionated colloidal organic carbon dynamics in a redox-controlled wetland. Geoderma. 2023;439:116667.

[39] Rod KA, Patel KF, Kumar S, Cantando E, Leng W, Kukkadapu RK, et al. Dispersible colloid facilitated release of organic carbon from two contrasting riparian sediments. Frontiers in Water. 2020;2:560707.

[40] Muller KA, Jiang P, Hammond G, Ahmadullah T, Song H-S, Kukkadapu R, et al. Lambda-PFLOTRAN 1.0: Workflow for Incorporating Organic Matter Chemistry Informed by Ultra High Resolution Mass Spectrometry into Biogeochemical Modeling. Geoscientific Model Development Discussions. 2024;2024:1–18.

[41] Service UNRC. Web soil survey 2023 [cited Retrieved November 7, 2019 for Beaver Creek and November 22, 2023 for Old Woman Creek, from]. https://websoilsurvey.nrcs.usda.gov/app/WebSoilSurvey.aspx.

[42] (NERRS) NNERRS. System-wide Monitoring Program. Data accessed from the NOAA NERRS Centralized Data Management Office [October 2022]. http://www.nerrsdata.org.

[43] Regier P, Ward ND, Indivero J, Wiese Moore C, Norwood M, Myers-Pigg A. Biogeochemical control points of connectivity between a tidal creek and its floodplain. Limnology & Oceanography: Letters. 2021. doi: 10.1002/lol2.10183

[44] Yabusaki SB, Myers‐Pigg AN, Ward ND, others. Floodplain inundation and salinization from a recently restored first‐order tidal stream. Water Resources Research. 2020;56(e2019WR026850). doi: 10.1029/2019WR026850

[45] Sawakuchi HO, Bastviken D, Sawakuchi AO, Ward ND, Borges CD, Tsai SM, et al. Oxidative mitigation of aquatic methane emissions in large Amazonian rivers. Glob Chang Biol. 2016;22(3):1075–85. doi: 10.1111/gcb.13169 26872424

[46] Ward ND, Indivero J, Gunn C, Wang W, Bailey V, McDowell NG. Longitudinal gradients in tree stem greenhouse gas concentrations across six Pacific Northwest coastal forests. J Geophys Res Biogeosci. 2019. doi: 10.1029/2019JG005064

[47] Wiesenburg DA, Guinasso NL Jr. Equilibrium solubilities of methane, carbon monoxide, and hydrogen in water and sea water. J Chem Eng Data. 1979;24(4):356–60.

[48] Magen C, Lapham LL, Pohlman JW, Marshall K, Bosman S, Casso M, et al. A simple headspace equilibration method for measuring dissolved methane. Limnology and Oceanography: Methods. 2014;12(9):637–50.

[49] Rounds S. Alkalinity and acid neutralizing capacity. US Geological Survey TWRI Book. 2001.

[50] Stookey LL. Ferrozine—a new spectrophotometric reagent for iron. Anal Chem. 1970;42(7):779–81.

[51] Dittmar T, Koch B, Hertkorn N, Kattner G. A simple and efficient method for the solid-phase extraction of dissolved organic matter (SPE-DOM) from seawater. Limnol Oceanogr Methods. 2008;6(6):230–5.

[52] Orton DJ, Tfaily MM, Moore RJ, LaMarche BL, Zheng X, Fillmore TL, et al. A customizable flow injection system for automated, high throughput, and time sensitive ion mobility spectrometry and mass spectrometry measurements. Anal Chem. 2018;90(1):737–44. doi: 10.1021/acs.analchem.7b02986 29161511 PMC5764703

[53] Tolić N, Liu Y, Liyu A, Shen Y, Tfaily MM, Kujawinski EB, et al. Formularity: software for automated formula assignment of natural and other organic matter from ultrahigh-resolution mass spectra. Anal Chem. 2017;89(23):12659–65. doi: 10.1021/acs.analchem.7b03318 29120613

[54] Team RC. R: A language and environment for statistical computing. R Foundation for Statistical Computing. (No Title). 2013.

[55] Bruland G, Richardson C. Spatial variability of soil properties in created, restored, and paired natural wetlands. Soil Science Society of America Journal. 2005;69(1):273–84.

[56] Sengupta A, Stegen JC, Bond-Lamberty B, Rivas-Ubach A, Zheng J, Handakumbura PP, et al. Antecedent conditions determine the biogeochemical response of coastal soils to seawater exposure. Soil Biology and Biochemistry. 2021;153:108104.

[57] Liu C-G, Xue C, Lin Y-H, Bai F-W. Redox potential control and applications in microaerobic and anaerobic fermentations. Biotechnol Adv. 2013;31(2):257–65. doi: 10.1016/j.biotechadv.2012.11.005 23178703

[58] Thompson A, Chadwick OA, Boman S, Chorover J. Colloid mobilization during soil iron redox oscillations. Environ Sci Technol. 2006;40(18):5743–9. doi: 10.1021/es061203b 17007135

[59] Dassonville F, Renault P. Interactions between microbial processes and geochemical transformations under anaerobic conditions: a review. Agronomie. 2002;22(1):51–68.

[60] Ponnamperuma FN. The chemistry of submerged soils. Advances in agronomy. 1972;24:29–96.

[61] Thompson A, Chadwick OA, Rancourt DG, Chorover J. Iron-oxide crystallinity increases during soil redox oscillations. Geochim Cosmochim Acta. 2006;70(7):1710–27.

[62] Lindsay WL. Chemical equilibria in soils1981.

[63] Grybos M, Davranche M, Gruau G, Petitjean P, Pédrot M. Increasing pH drives organic matter solubilization from wetland soils under reducing conditions. Geoderma. 2009;154(1–2):13–9.

[64] Van Breemen N. Effects of redox processes on soil acidity. Netherlands Journal of Agricultural Science. 1987;35(3):271–9.

[65] Patel KF, Rod KA, Zheng J, Regier P, Machado-Silva F, Bond-Lamberty B, et al. Time to anoxia: Observations and predictions of oxygen drawdown following coastal flood events. Geoderma. 2024;444:116854.

[66] Bergelin A, Van Hees P, Wahlberg O, Lundström U. The acid–base properties of high and low molecular weight organic acids in soil solutions of podzolic soils. Geoderma. 2000;94(2–4):223–35.

[67] Zhao Q, Dunham-Cheatham S, Adhikari D, Chen C, Patel A, Poulson SR, et al. Oxidation of soil organic carbon during an anoxic-oxic transition. Geoderma. 2020;377:114584.

[68] Reddy K, Patrick W Jr. Effect of alternate aerobic and anaerobic conditions on redox potential, organic matter decomposition and nitrogen loss in a flooded soil. Soil Biology and Biochemistry. 1975;7(2):87–94.

[69] Yan J, Lazouskaya V, Jin Y. Soil colloid release affected by dissolved organic matter and redox conditions. Vadose Zone Journal. 2016;15(3):vzj2015. 02.0026.

[70] Bhattacharyya A, Campbell AN, Tfaily MM, Lin Y, Kukkadapu RK, Silver WL, et al. Redox Fluctuations Control the Coupled Cycling of Iron and Carbon in Tropical Forest Soils. Environ Sci Technol. 2018;52(24):14129–39. doi: 10.1021/acs.est.8b03408 30451506

[71] Marschner P. Processes in submerged soils–linking redox potential, soil organic matter turnover and plants to nutrient cycling. Plant and Soil. 2021;464(1):1–12.

[72] Kim HS, Pfaender FK. Effects of microbially mediated redox conditions on PAH− soil interactions. Environ Sci Technol. 2005;39(23):9189–96. doi: 10.1021/es0508976 16382941

[73] Kida M, Tomotsune M, Iimura Y, Kinjo K, Ohtsuka T, Fujitake N. High salinity leads to accumulation of soil organic carbon in mangrove soil. Chemosphere. 2017;177:51–5. doi: 10.1016/j.chemosphere.2017.02.074 28282623

[74] Dunn C, Freeman C. The role of molecular weight in the enzyme-inhibiting effect of phenolics: the significance in peatland carbon sequestration. Ecol Eng. 2018;114:162–6.

[75] Morrissey EM, Gillespie JL, Morina JC, Franklin RB. Salinity affects microbial activity and soil organic matter content in tidal wetlands. Glob Chang Biol. 2014;20(4):1351–62. doi: 10.1111/gcb.12431 24307658

[76] Dou F, Ping CL, Guo L, Jorgenson T. Estimating the impact of seawater on the production of soil water‐extractable organic carbon during coastal erosion. J Environ Qual. 2008;37(6):2368–74. doi: 10.2134/jeq2007.0403 18948491

[77] Kleber M, Bourg IC, Coward EK, Hansel CM, Myneni SC, Nunan N. Dynamic interactions at the mineral–organic matter interface. Nature Reviews Earth & Environment. 2021;2(6):402–21.

[78] Tomaszewski E, Coward E, Sparks D. Ionic strength and species drive iron–carbon adsorption dynamics: implications for carbon cycling in future coastal environments. Environmental Science & Technology Letters. 2021;8(8):719–24.

[79] Wang Y, Wang H, He J-S, Feng X. Iron-mediated soil carbon response to water-table decline in an alpine wetland. Nat Commun. 2017;8:15972. doi: 10.1038/ncomms15972 28649988 PMC5490263

[80] Wagai R, Mayer LM. Sorptive stabilization of organic matter in soils by hydrous iron oxides. Geochim Cosmochim Acta. 2007;71(1):25–35.

[81] Buettner SW, Kramer MG, Chadwick OA, Thompson A. Mobilization of colloidal carbon during iron reduction in basaltic soils. Geoderma. 2014;221:139–45.

[82] Chen C, Hall SJ, Coward E, Thompson A. Iron-mediated organic matter decomposition in humid soils can counteract protection. Nature communications. 2020;11(1):2255. doi: 10.1038/s41467-020-16071-5 32382079 PMC7206102

[83] Villa JA, Ju Y, Stephen T, Rey‐Sanchez C, Wrighton KC, Bohrer G. Plant‐mediated methane transport in emergent and floating‐leaved species of a temperate freshwater mineral‐soil wetland. Limnology and Oceanography. 2020;65(7):1635–50.

[84] Bianchi TS, Wysocki LA, Stewart M, Filley TR, McKee BA. Temporal variability in terrestrially-derived sources of particulate organic carbon in the lower Mississippi River and its upper tributaries. Geochim Cosmochim Acta. 2007;71(18):4425–37.

[85] Liu M, Ussiri DA, Lal R. Soil organic carbon and nitrogen fractions under different land uses and tillage practices. Communications in Soil Science and Plant Analysis. 2016;47(12):1528–41.

[86] Qi Y, Xie Q, Wang J-J, He D, Bao H, Fu Q-L, et al. Deciphering dissolved organic matter by Fourier transform ion cyclotron resonance mass spectrometry (FT-ICR MS): from bulk to fractions and individuals. Carbon Research. 2022;1(1):3.

[87] Stagg CL, Baustian MM, Perry CL, Carruthers TJ, Hall CT. Direct and indirect controls on organic matter decomposition in four coastal wetland communities along a landscape salinity gradient. J Ecol. 2018;106(2):655–70.

[88] Neubauer S, Franklin R, Berrier D. Saltwater intrusion into tidal freshwater marshes alters the biogeochemical processing of organic carbon. Biogeosciences. 2013;10(12):8171–83.

[89] Qu W, Li J, Han G, Wu H, Song W, Zhang X. Effect of salinity on the decomposition of soil organic carbon in a tidal wetland. Journal of Soils and Sediments. 2019;19:609–17.

[90] Zhang J, Wang JJ, Xiao R, Deng H, DeLaune RD. Effect of salinity on greenhouse gas production and emission in marsh soils during the decomposition of wetland plants. Journal of Soils and Sediments. 2023;23(1):131–44.

[91] Stagg CL, Schoolmaster DR, Krauss KW, Cormier N, Conner WH. Causal mechanisms of soil organic matter decomposition: deconstructing salinity and flooding impacts in coastal wetlands. Ecology. 2017;98(8):2003–18. doi: 10.1002/ecy.1890 28489250

[92] Colombo C, Palumbo G, He J-Z, Pinton R, Cesco S. Review on iron availability in soil: interaction of Fe minerals, plants, and microbes. Journal of soils and sediments. 2014;14:538–48.

[93] Borch T, Kretzschmar R, Kappler A, Cappellen PV, Ginder-Vogel M, Voegelin A, et al. Biogeochemical redox processes and their impact on contaminant dynamics. Environ Sci Technol. 2010;44(1):15–23. doi: 10.1021/es9026248 20000681

[94] Van Breemen N. Redox processes of iron and sulfur involved in the formation of acid sulfate soils. Iron in soils and clay minerals: Springer; 1988. p. 825–41.

[95] Joshi P, ThomasArrigo LK, Sawwa D, Sauter L, Kappler A. Complexation by Particulate Organic Matter Alters Iron Redox Behavior. ACS Earth and Space Chemistry. 2024;8(2):310–22.

[96] Kukkadapu RK, Zachara JM, Fredrickson JK, Kennedy DW. Biotransformation of two-line silica-ferrihydrite by a dissimilatory Fe (III)-reducing bacterium: formation of carbonate green rust in the presence of phosphate. Geochim Cosmochim Acta. 2004;68(13):2799–814.

[97] Tully K, Gedan K, Epanchin-Niell R, Strong A, Bernhardt ES, BenDor T, et al. The Invisible Flood: The Chemistry, Ecology, and Social Implications of Coastal Saltwater Intrusion. Bioscience. 2019;69(5):368–78. doi: 10.1093/biosci/biz027

[98] Wuebbles D, Cardinale B, Cherkauer K, Davidson-Arnott R, Hellmann J, Infante D, et al. An assessment of the impacts of climate change on the Great Lakes. Environmental law & policy center. 2019;104(3–4):629–52.

[99] Lønborg C, Carreira C, Jickells T, Álvarez-Salgado XA. Impacts of global change on ocean dissolved organic carbon (DOC) cycling. Frontiers in Marine Science. 2020;7:466.

[100] Smith AJ, McGlathery K, Chen Y, Ewers Lewis CJ, Doney SC, Gedan K, et al. Compensatory mechanisms absorb regional carbon losses within a rapidly shifting coastal mosaic. Ecosystems. 2024;27(1):122–36.

[101] Clark JM, Lane SN, Chapman PJ, Adamson JK. Export of dissolved organic carbon from an upland peatland during storm events: Implications for flux estimates. Journal of Hydrology. 2007;347(3–4):438–47.

[102] Altor AE, Mitsch WJ. Pulsing hydrology, methane emissions and carbon dioxide fluxes in created marshes: A 2-year ecosystem study. Wetlands. 2008;28(2):423–38.

[103] Noe GB, Krauss KW, Lockaby BG, Conner WH, Hupp CR. The effect of increasing salinity and forest mortality on soil nitrogen and phosphorus mineralization in tidal freshwater forested wetlands. Biogeochemistry. 2013;114:225–44.

[104] Regier PJ, Ward ND, Myers‐Pigg AN, Grate J, Freeman MJ, Ghosh RN. Seasonal drivers of dissolved oxygen across a tidal creek–marsh interface revealed by machine learning. Limnology and Oceanography. 2023;68(10):2359–74.

[105] Baustian MM, Stagg CL, Perry CL, Moss LC, Carruthers TJB, Allison M. Relationships Between Salinity and Short-Term Soil Carbon Accumulation Rates from Marsh Types Across a Landscape in the Mississippi River Delta. Wetlands. 2017;37(2):313–24. doi: 10.1007/s13157-016-0871-3

[106] Moomaw WR, Chmura G, Davies GT, Finlayson C, Middleton BA, Natali SM, et al. Wetlands in a changing climate: science, policy and management. Wetlands. 2018;38(2):183–205.

[107] Villa JA, Bernal B. Carbon sequestration in wetlands, from science to practice: An overview of the biogeochemical process, measurement methods, and policy framework. Ecol Eng. 2018;114:115–28.

[108] Wang F, Lu X, Sanders CJ, Tang J. Tidal wetland resilience to sea level rise increases their carbon sequestration capacity in United States. Nature Communications. 2019;10(1):5434. doi: 10.1038/s41467-019-13294-z 31780651 PMC6883032

