# Supplementary material for: Interactive effects of salinity, redox, and colloids on greenhouse gas production and carbon mobility in coastal wetland soils
Source: PLoS One. 2024 Dec 30;19(12):e0316341. doi: 10.1371/journal.pone.0316341 (PMC11684665; doi:10.1371/journal.pone.0316341)
Supplement: S1 Text — The file “S1 Text.pdf” contains additional data visualizations and summaries. (PDF) [file pone.0316341.s001.pdf]

Supporting Information – S1 Text

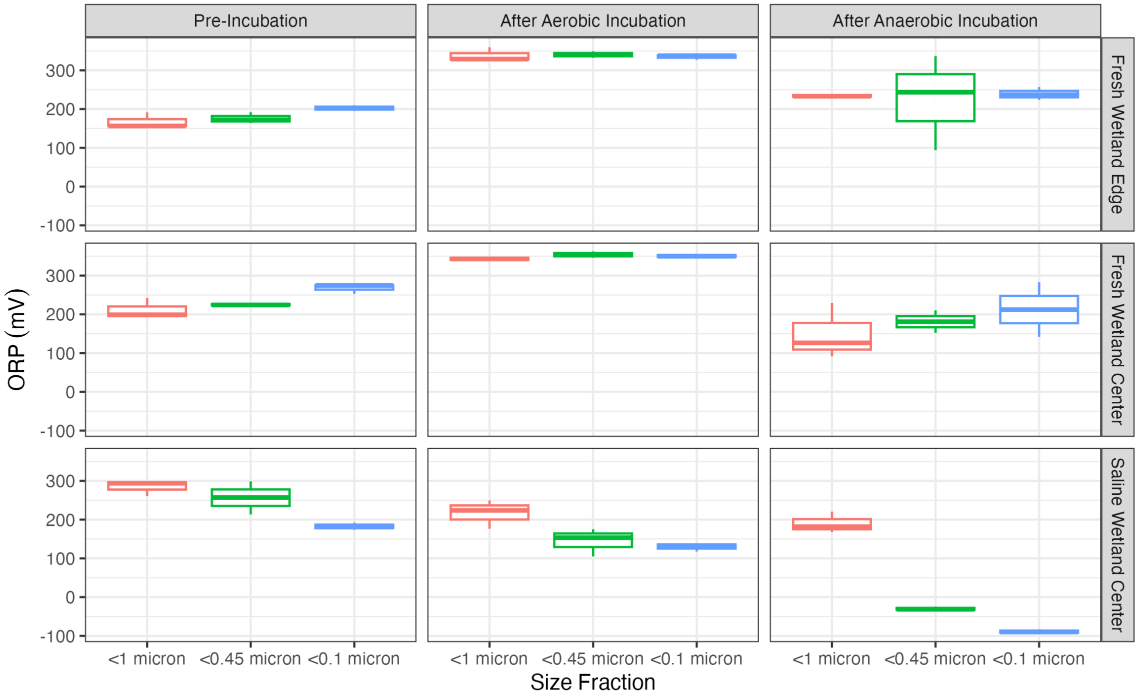

Figure 1. Oxidation-reduction potential (of size-fractionated and unfiltered incubates before and after incubations. It did not make conceptual sense to calculate the contribution of each size fraction via subtraction. Thus, we show data from the independently centrifuged < 0.1 µm, <0.45 µm, and <1 µm fractions.

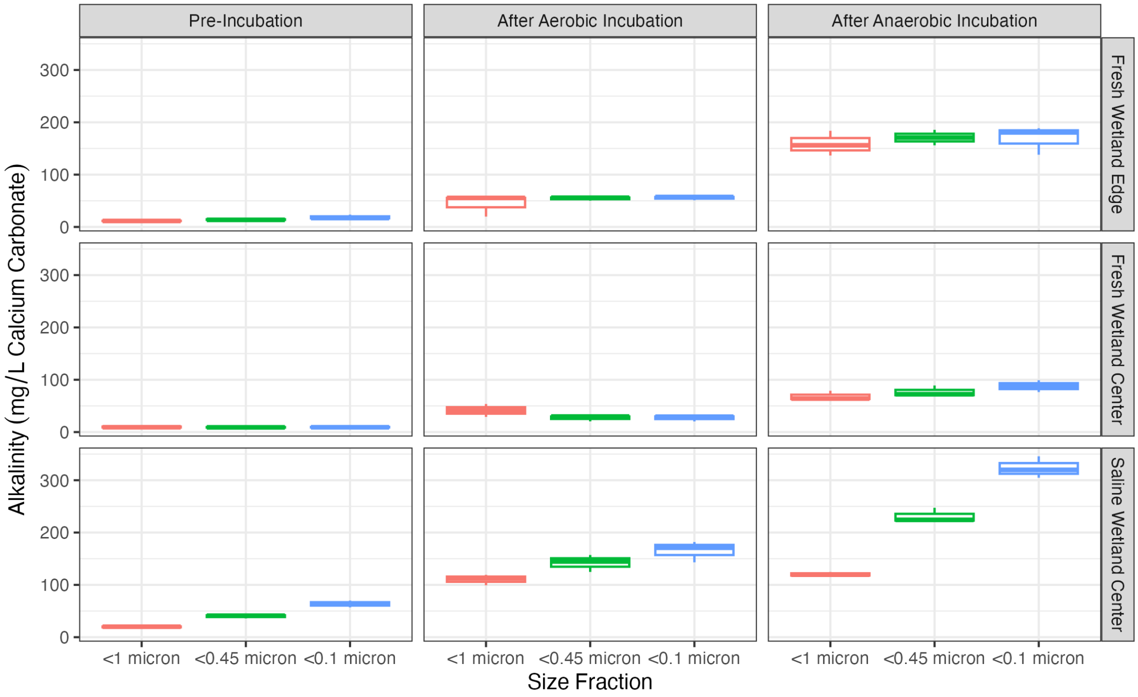

Figure 2. Alkalinity of size-fractionated and unfiltered incubates before and after incubations. It did not make conceptual sense to calculate the contribution of each size fraction via subtraction. Thus, we show data from the independently centrifuged < 0.1 µm, <0.45 µm, and <1 µm fractions.

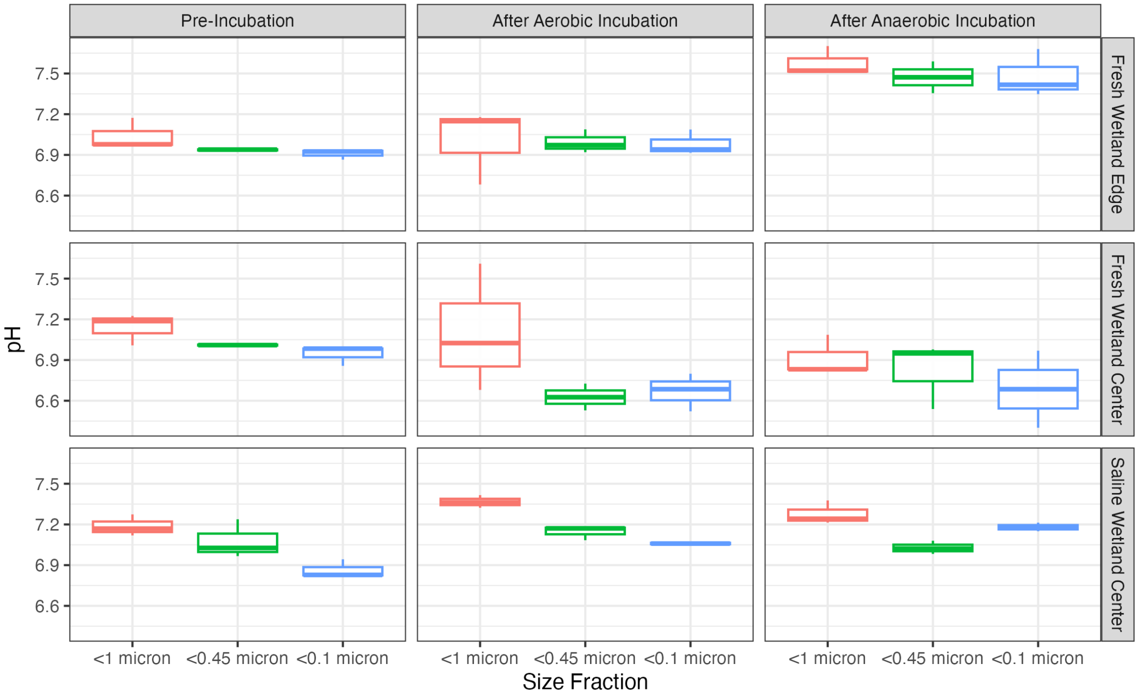

Figure 3. pH of size-fractionated and unfiltered incubates before and after incubations. It did not make conceptual sense to calculate the contribution of each size fraction via subtraction. Thus, we show data from the independently centrifuged < 0.1 µm, <0.45 µm, and <1 µm fractions.

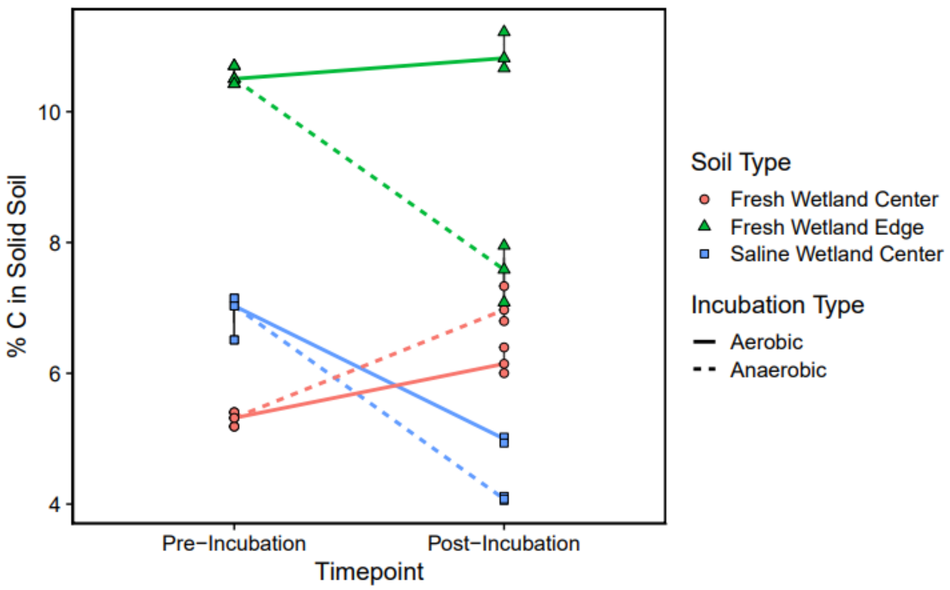

Figure 4. Percent carbon in solid soils pre- and post-incubation.

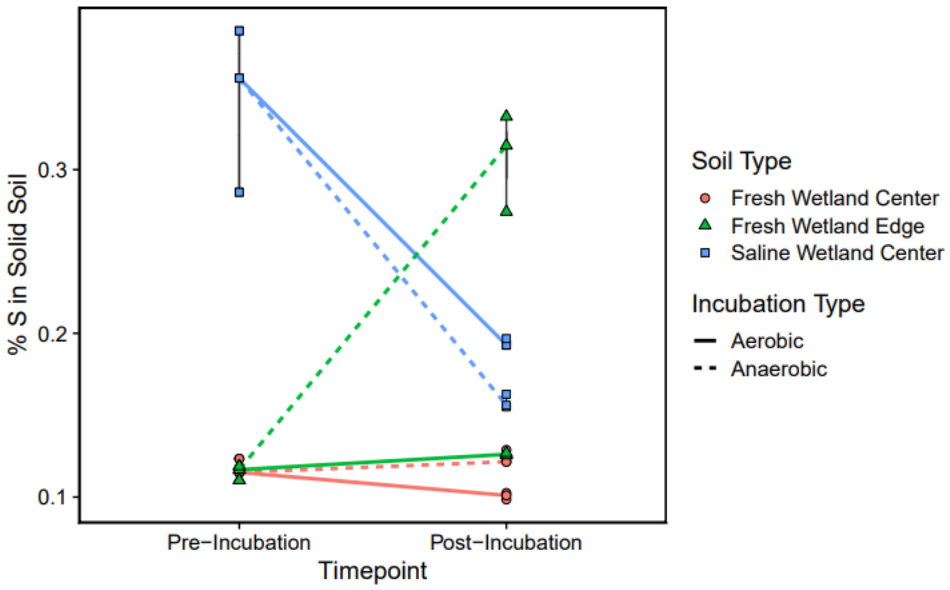

Figure 5. Percent sulfur in solid soils pre- and post-incubation.

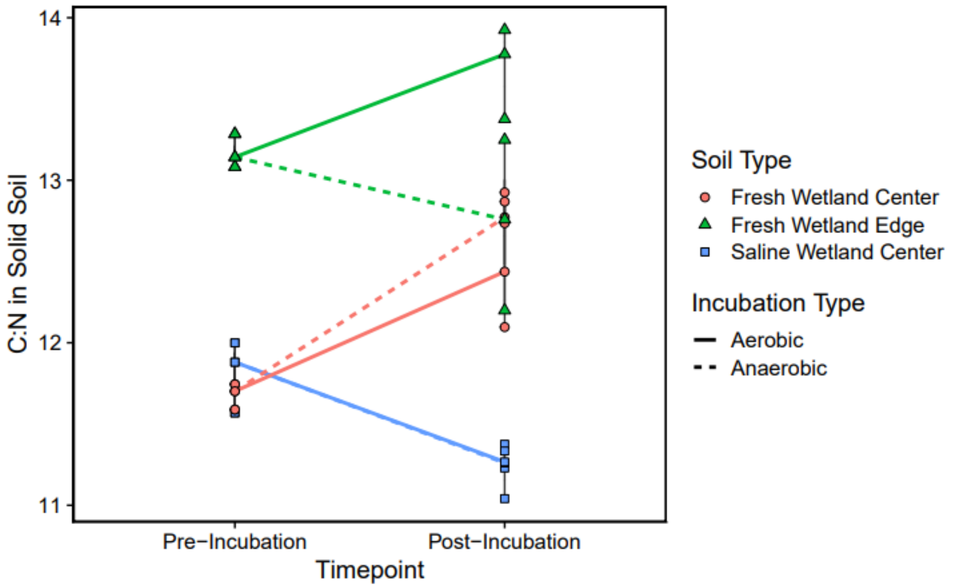

Figure 6. Carbon to nitrogen ratios in solid soils pre- and post-incubation

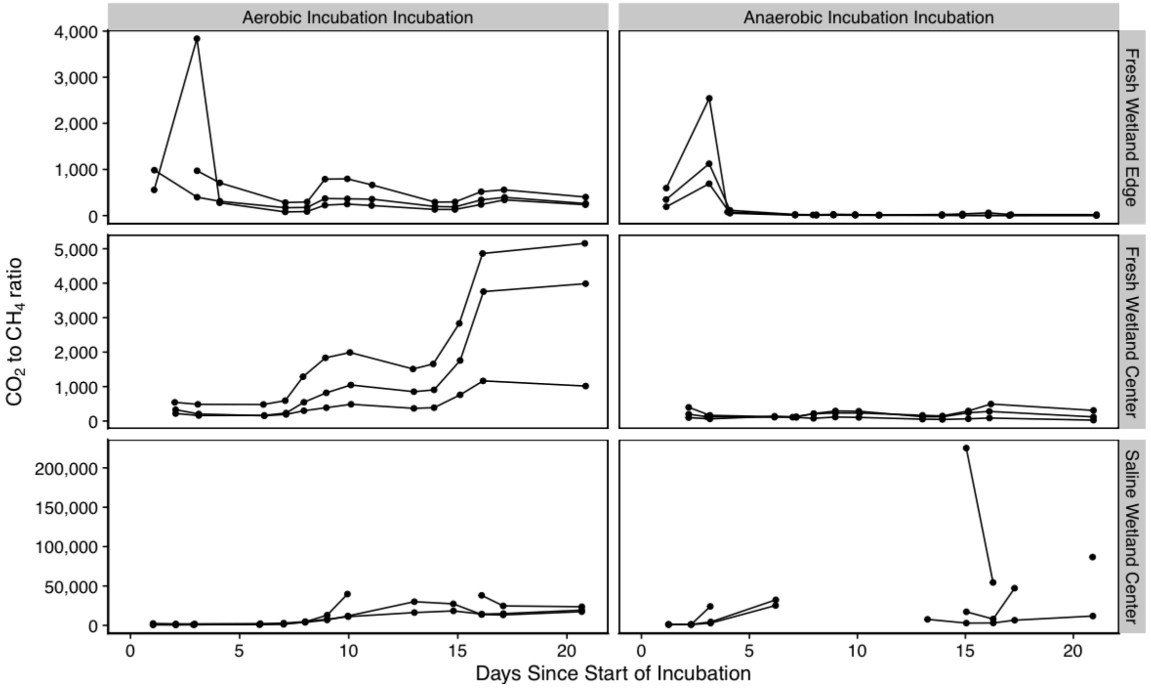

Figure 7. The molar ratio of CO_2 to CH_4 (per kg of dry soil) throughout the incubations. Note that y-axis scaling varies between top, middle, and bottom panels to allow trends to be seen more clearly. Missing values occurred when CH_4 concentrations were zero.

Soil/Incubation Type	FW-E Pre-Incubation	FW-E Aerobic Incubation	FW-E Anaerobic Incubation	FW-C Pre-Incubation	FW-C Aerobic Incubation	FW-C Anaerobic Incubation	SW-C Pre-Incubation	SW-C Aerobic Incubation	SW-C Anaerobic Incubation
**Compound Class**									
Amino Sugars	5 ± 2	3 ± 0	2 ± 0	5 ± 1	3 ± 1	3 ± 1	5 ± 0	5 ± 0	5 ± 0
Carbohydrates	3 ± 2	2 ± 1	1 ± 1	3 ± 2	2 ± 1	1 ± 0	3 ± 0	2 ± 0	2 ± 1
Condensed Hydrocarbons	11 ± 4	18 ± 4	22 ± 3	12 ± 3	18 ± 2	20 ± 4	8 ± 1	9 ± 1	8 ± 0
Lignin	36 ± 5	49 ± 6	45 ± 2	38 ± 5	48 ± 2	44 ± 2	36 ± 2	30 ± 1	29 ± 2
Lipids	11 ± 3	4 ± 1	6 ± 1	9 ± 3	4 ± 1	7 ± 3	15 ± 1	19 ± 1	21 ± 3
Other	1 ± 0	0 ± 0	1 ± 0	1 ± 0	1 ± 0	1 ± 0	1 ± 0	0 ± 0	0 ± 0
Proteins	25 ± 6	13 ± 1	12 ± 3	22 ± 6	13 ± 1	13 ± 4	22 ± 1	25 ± 1	26 ± 1
Tannins	7 ± 3	11 ± 2	12 ± 2	8 ± 4	11 ± 2	11 ± 3	8 ± 0	7 ± 0	6 ± 1
Unsaturated Hydrocarbons	1 ± 1	1 ± 0	0 ± 0	2 ± 1	1 ± 0	1 ± 0	2 ± 0	2 ± 0	3 ± 0
Number of Peaks	8,692 ± 2,339	10,340 ± 1,584	8,894 ± 1,485	9,697 ± 2,052	10,370 ± 2,559	8,687 ± 1,454	8,924 ± 390	7,067 ± 536	6,770 ± 846

Table 1. Summary of the total number of peaks detected via FT-ICR-MS and the calculated percent contribution of different compound classes to the portion of the TOC pool captured within the analytical window. The largest < 1 μm size fraction is presented here.

Soil/Incubation Type	FW-E Pre-Incubation	FW-E Aerobic Incubation	FW-E Anaerobic Incubation	FW-C Pre-Incubation	FW-C Aerobic Incubation	FW-C Anaerobic Incubation	SW-C Pre-Incubation	SW-C Aerobic Incubation	SW-C Anaerobic Incubation
**Compound Class**									
Amino Sugars	6 ± 1	3 ± 0	2 ± 0	5 ± 1	3 ± 1	2 ± 1	5 ± 0	5 ± 0	5 ± 0
Carbohydrates	4 ± 1	2 ± 1	1 ± 1	3 ± 2	2 ± 1	1 ± 0	4 ± 0	2 ± 0	2 ± 0
Condensed Hydrocarbons	13 ± 5	17 ± 3	20 ± 4	14 ± 4	19 ± 2	21 ± 3	12 ± 3	10 ± 1	11 ± 1
Lignin	38 ± 1	50 ± 5	45 ± 1	39 ± 3	48 ± 2	45 ± 2	41 ± 2	34 ± 1	31 ± 1
Lipids	9 ± 2	4 ± 0	7 ± 3	8 ± 2	5 ± 1	6 ± 2	9 ± 2	16 ± 1	19 ± 1
Other	1 ± 0	1 ± 0	1 ± 0	1 ± 0	1 ± 0	1 ± 0	1 ± 0	0 ± 0	0 ± 0
Proteins	21 ± 4	12 ± 1	13 ± 4	20 ± 5	12 ± 1	11 ± 3	18 ± 3	23 ± 1	24 ± 1
Tannins	8 ± 3	10 ± 1	11 ± 3	9 ± 3	11 ± 2	12 ± 2	10 ± 2	7 ± 1	7 ± 1
Unsaturated Hydrocarbons	1 ± 1	1 ± 0	1 ± 0	1 ± 1	1 ± 0	1 ± 0	1 ± 0	2 ± 0	2 ± 0
Number of Peaks	10,622 ± 2,131	10,517 ± 2,086	8,342 ± 1,916	9,864 ± 2,327	10,427 ± 2,554	8,784 ± 1,479	9,645 ± 444	8,055 ± 675	6,833 ± 312

Table 2. Summary of the total number of peaks detected via FT-ICR-MS and the calculated percent contribution of different compound classes to the portion of the TOC pool captured within the analytical window. The medium < 0.45 µm size fraction is presented here.

Soil/Incubation Type	FW-E Pre-Incubation	FW-E Aerobic Incubation	FW-E Anaerobic Incubation	FW-C Pre-Incubation	FW-C Aerobic Incubation	FW-C Anaerobic Incubation	SW-C Pre-Incubation	SW-C Aerobic Incubation	SW-C Anaerobic Incubation
**Compound Class**									
Amino Sugars	4 ± 1	3 ± 1	2 ± 0	4 ± 1	2 ± 1	2 ± 0	3 ± 1	3 ± 0	3 ± 0
Carbohydrates	3 ± 2	2 ± 0	1 ± 0	2 ± 2	1 ± 1	1 ± 0	2 ± 0	2 ± 0	2 ± 0
Condensed Hydrocarbons	15 ± 5	19 ± 5	24 ± 1	15 ± 5	21 ± 3	24 ± 1	21 ± 2	19 ± 0	19 ± 0
Lignin	40 ± 1	49 ± 4	46 ± 1	40 ± 4	48 ± 2	46 ± 2	44 ± 1	40 ± 0	37 ± 2
Lipids	8 ± 2	4 ± 1	4 ± 0	8 ± 3	4 ± 1	4 ± 1	4 ± 1	8 ± 0	10 ± 1
Other	1 ± 0	0 ± 0	0 ± 0	1 ± 0	0 ± 0	0 ± 0	0 ± 0	0 ± 0	0 ± 0
Proteins	19 ± 4	11 ± 2	10 ± 0	20 ± 7	10 ± 2	10 ± 1	11 ± 2	15 ± 1	16 ± 1
Tannins	8 ± 3	11 ± 2	12 ± 1	8 ± 4	12 ± 2	13 ± 1	13 ± 1	11 ± 0	10 ± 0
Unsaturated Hydrocarbons	1 ± 1	1 ± 0	0 ± 0	1 ± 1	1 ± 0	0 ± 0	0 ± 0	1 ± 0	1 ± 0
Number of Peaks	10,683 ± 578	10,708 ± 1,431	9,083 ± 1,473	9,538 ± 2,267	10,373 ± 2,479	8,936 ± 1,762	10,495 ± 326	8,805 ± 491	8,074 ± 640

Table 3. Summary of the total number of peaks detected via FT-ICR-MS and the calculated percent contribution of different compound classes to the portion of the TOC pool captured within the analytical window. The smallest < 0.1 μm size fraction is presented here.
